# Structural and genetic basis of HIV-1 envelope V2 apex recognition by rhesus broadly neutralizing antibodies

**DOI:** 10.1084/jem.20250638

**Published:** 2025-08-18

**Authors:** Ryan S. Roark, Rumi Habib, Jason Gorman, Hui Li, Andrew Jesse Connell, Mattia Bonsignori, Yicheng Guo, Michael P. Hogarty, Adam S. Olia, Kirsten J. Sowers, Baoshan Zhang, Frederic Bibollet-Ruche, Tatsiana Bylund, Sean Callaghan, John W. Carey, Gabriele Cerutti, Darcy R. Harris, Wanting He, Emily Lewis, Tracy Liu, Rosemarie D. Mason, Yujie Qiao, Younghoon Park, Juliette M. Rando, Ajay Singh, Jeremy J. Wolff, Q. Paula Lei, Mark K. Louder, Raiees Andrabi, Nicole A. Doria-Rose, Kevin O. Saunders, Michael S. Seaman, Barton F. Haynes, Daniel W. Kulp, John R. Mascola, Mario Roederer, Theodore C. Pierson, Zizhang Sheng, Beatrice H. Hahn, George M. Shaw, Peter D. Kwong, Lawrence Shapiro

**Affiliations:** 1 Aaron Diamond AIDS Research Center, Columbia University Vagelos College of Physicians and Surgeons, New York, NY, USA; 2 Department of Biochemistry and Molecular Biophysics, Columbia University, New York, NY, USA; 3 Zuckerman Mind Brain Behavior Institute, Columbia University, New York, NY, USA; 4 Departments of Medicine and Microbiology, Perelman School of Medicine, University of Pennsylvania, Philadelphia, PA, USA; 5 Vaccine and Immunotherapy Center, The Wistar Institute, Philadelphia, PA, USA; 6 Vaccine Research Center, National Institute of Allergy and Infectious Diseases, National Institutes of Health, Bethesda, MD, USA; 7 Division of Viral Products, Center for Biologics Evaluation and Research, Food and Drug Administration, Silver Spring, MD, USA; 8 Translational Immunobiology Unit, Laboratory of Infectious Diseases, National Institute of Allergy and Infectious Diseases, National Institutes of Health, Bethesda, MD, USA; 9 Department of Immunology and Microbiology, The Scripps Research Institute, La Jolla, CA, USA; 10 Department of Integrative Immunobiology, Duke University School of Medicine, Durham, NC, USA; 11 Duke Human Vaccine Institute, Duke University School of Medicine, Durham, NC, USA; 12 Department of Surgery, Duke University School of Medicine, Durham, NC, USA; 13 Department of Molecular Genetics and Microbiology, Duke University School of Medicine, Durham, NC, USA; 14 Center for Virology and Vaccine Research, Beth Israel Deaconess Medical Center, Boston, MA, USA; 15 Department of Medicine, Duke University School of Medicine, Durham, NC, USA

## Abstract

Broadly neutralizing antibodies targeting the V2 apex of HIV-1 envelope are desired as vaccine design templates, but few have been described. Here, we report 11 lineages of V2 apex–neutralizing antibodies from simian-human immunodeficiency virus (SHIV)-infected rhesus macaques and determine cryo-EM structures for 9. A single V2 apex–neutralizing lineage accounted for cross-clade breadth in most macaques, and somatic hypermutation relative to breadth was generally low, exemplified by antibody V033-a.01 with <5% nucleotide mutation and 37% breadth (208-strain panel). Envelope complex structures revealed eight different antibody classes (one multi-donor) and the complete repertoire of all five possible recognition topologies, recapitulating canonical human modes of apex insertion and C-strand hydrogen bonding. Despite this diversity in recognition, all rhesus–V2 apex antibodies were derived from reading frame two of the DH3-15*01 gene. Collectively, these results define—in rhesus—the structural and genetic basis of HIV-1 V2 apex recognition and demonstrate unprecedented structural plasticity of a highly selected immunogenetic element.

## Teaser

Roark et al. infect macaques with chimeric simian-human immunodeficiency viruses to induce broad HIV-1-neutralizing antibodies targeting the V2 apex site-of-vulnerability. From 10 infected macaques, 11 V2 apex-directed antibody lineages were identified, and 9 antibody-antigen complexes were characterized, collectively defining both structural repertoire and genetic basis for V2 apex recognition.

## Introduction

Antibodies directed to the V2 apex of the HIV-1 envelope trimer (Env) comprise one of the most desired categories of broadly neutralizing antibodies elicited by natural infection that HIV-1 vaccine developers have targeted for re-elicitation through antibody-templated vaccine approaches (Kwong and Mascola, 2012; Burton and Hangartner, 2016; Kwong et al., 2017; Medina-Ramírez et al., 2017; Willis et al., 2022; Haynes et al., 2023). First, broadly neutralizing antibodies targeting the V2 apex are among the most common broadly neutralizing specificities elicited in HIV-1–infected humans and simian-human immunodeficiency virus (SHIV)-infected rhesus macaques (Walker et al., 2010; Landais and Moore, 2018; Roark et al., 2021; Habib et al., 2025, *Preprint*). Second, the V2 apex of HIV-1 is conserved structurally, functionally, and antigenically across multiple HIV-1 subtypes and across a broad spectrum of primate lentiviruses (Mclellan et al., 2011; Julien et al., 2013; Barbian et al., 2015; Kwon et al., 2015; Andrabi et al., 2019; Bibollet-Ruche et al., 2023). Third, V2 apex broadly neutralizing antibodies can require less somatic hypermutation (SHM) to acquire breadth and potency compared with other epitope specificities (Chuang et al., 2019; Griffith and McCoy, 2021). On the negative side, to penetrate the apical glycan shield of Env and bind underlying conserved positively charged residues, broadly neutralizing V2 apex antibodies must contain heavy chain third complementarity-determining regions (HCDR3s) that are exceptionally long, anionic, and often tyrosine sulfated (Andrabi et al., 2015; Gorman et al., 2016). Only certain D genes may have sequence features consistent with these requirements (Briney et al., 2012). Moreover, long HCDR3s are generally encoded during the process of V-D-J recombination and non-templated nucleotide addition and may be disfavored because of autoreactivity or other factors related to checkpoint inhibition (Briney et al., 2012; Verkoczy and Diaz, 2014; Kelsoe and Haynes, 2017).

To date, only five human V2 apex broadly neutralizing antibody lineages—characterized by single antibody per trimer stoichiometry—have been isolated and defined structurally: the PG9/PG16 lineage (Walker et al., 2009), the CH01/CH03 lineage (Bonsignori et al., 2011), the PGT145/PGDM1400 lineage (Walker et al., 2011; Sok et al., 2014; Mason et al., 2025), the CAP256-VRC26 lineage (Doria-Rose et al., 2014; Doria-Rose et al., 2016), and the PCT64 lineage (Landais et al., 2017). The extended HCDR3s of these V2 apex neutralizers interact with Env (1) by inserting into a cationic hole at the trimer apex (PGT145/PGDM1400 and PCT64) (Lee et al., 2017; Rantalainen et al., 2018; Willis et al., 2022), (2) by mainchain hydrogen bonding to the C-strand at the hole edge (PG9/16 and CH01/CH03) (Mclellan et al., 2011; Pancera et al., 2013; Gorman et al., 2016), or (3) by a combined mode, both inserting and hydrogen bonding (CAP256-VRC26) (Gorman et al., 2020). However, of the five possible recognition topologies utilizing apex insertion and parallel or antiparallel C-strand hydrogen bonding, alone or in combination, only three topologies have been observed so far: apex insertion alone for PGT145 and PCT64; parallel strand hydrogen bonding for both PG9/16 and CH01 and antiparallel hydrogen bonding for CAP256-VRC26 in the combined mode. These findings beg the question: Could the full complement of all five possible recognition topologies be utilized for V2 apex recognition? And what types of genetic recombination and what levels of SHM enable V2 apex recognition? With only five example templates, the answers to these questions have been unclear.

The prevalence of appropriate HCDR3s within the naïve B cell population that might serve as precursors to V2 apex–directed broadly neutralizing antibodies has been investigated. Two factors appear critical: (1) generation of long, negatively charged HCDR3s by recombination, and (2) placement of appropriate HCDR3s into contexts that enable V2 apex recognition (Willis et al., 2016; Briney et al., 2012; Willis et al., 2022). For preclinical vaccine development, appropriate HCDR3s need to be generated in standard vaccine test species. Long HCDR3s are rare in mice, rats, and guinea pigs, which are often used to assess vaccine immunogens, whereas nonhuman primates can generate long HCDR3s (Morgan et al., 2008; Sundling et al., 2012; Francica et al., 2015; Hu et al., 2015) and may thus represent a more appropriate vaccine-test species.

Previously, we observed the induction of a V2 apex–directed lineage, RHA1 (Roark et al., 2021), from a macaque (RM5695) that had been infected with SHIV derived from the CH505 transmitter founder virus, which exhibited ∼50% neutralization breadth and a PGT145/PGDM1400 needle-like inserting mode of epitope recognition (Landais et al., 2017; Lee et al., 2017). We also identified specific HIV-1 strains, sensitive to neutralization by germline versions of V2 apex broadly neutralizing antibodies, either unmutated common ancestors or V gene reverted (gHgL) (Andrabi et al., 2015; Gorman et al., 2016). To obtain additional examples of V2 apex broadly neutralizing antibodies, we infected rhesus macaques with different SHIV strains—particularly by those that were sensitive to neutralization by germline or ancestor versions of V2 apex broadly neutralizing antibodies—and assessed the ability of these SHIVs to induce broad V2 apex–directed responses. With 10 of these SHIV-infected rhesus macaques, we used antigen-specific single-cell sorting to isolate V2 apex broadly neutralizing antibody lineages and to conduct a systematic analysis of their immunogenetics, neutralization phenotypes, complex structures, antibody classes, and topologies of recognition. For structural characterization, we used single-particle cryo-EM analysis of complexes between the antigen-binding fragments (Fab’s) of antibodies and Envs, stabilized in the prefusion-closed conformation. Notably, we observed a single rhesus D gene to provide the full complement of recognition topologies—with the same D-gene–encoded amino acids inserting into a hole at the trimer apex, hydrogen bonding to an exposed strand, or forming part of a loop scaffold—thereby demonstrating how a highly selected genetic element can nevertheless play divergent structural roles. Overall, the data presented in this study nearly triples the number of known cross-clade–neutralizing V2 apex antibody lineages and their co-complex structures.

## Results

### Single-cell sorting identifies 11 V2 apex cross-clade–neutralizing lineages from rhesus macaques

**Figure 1.**
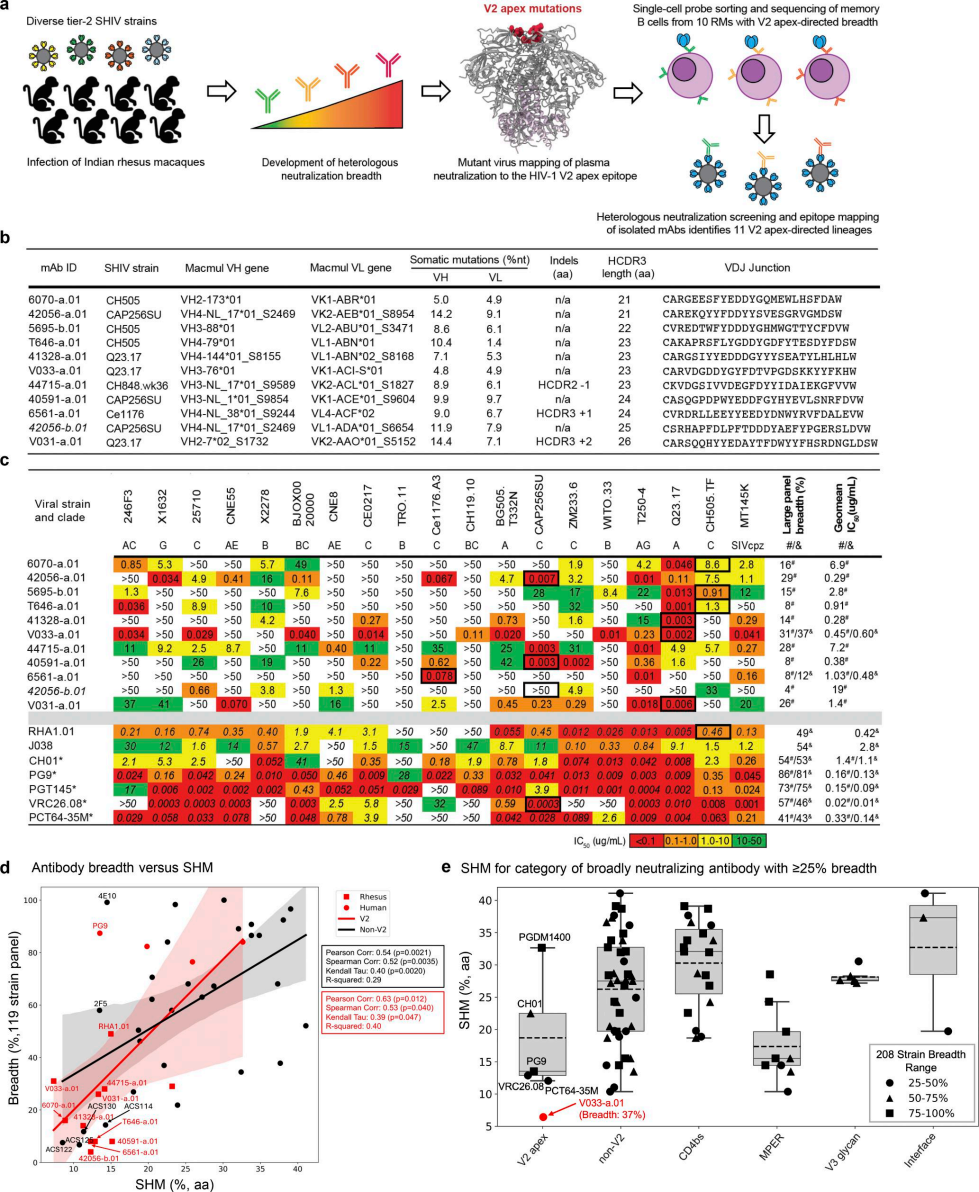
**Single-cell sorting identifies 11 cross-clade–neutralizing lineages from 10 SHIV-infected rhesus macaques with HIV-1 V2 apex–directed heterologous neutralization breadth. (a)** Schematic for the present study. **(b)** Macaque host ID, infecting SHIV strain, and immunogenetics of a representative mAb from each of the 11 rhesus lineages reported in this study. **(c)** Neutralization breadth and potency of representative monoclonal lineage antibodies. Left: Neutralization activity against a 19-member panel of cross-clade tier-2 HIV-1 strains and a simian immunodeficiency virus infecting chimpanzees (SIVcpz), MT145K, which has been shown to bear the conserved HIV-1 V2 apex epitope. Data are reported as IC_50_ titer (µg/ml) and colored according to the legend. Bold boxes demarcate activity against autologous virus; for example, 6070-a.01 was isolated from an animal infected with an SHIV bearing the HIV-1 CH505.TF Env (SHIV.CH505). All small panel neutralization experiments were performed in duplicate and repeated twice. Right: Neutralization breadth and geomean IC_50_ against one or two large cross-clade panels of HIV-1 strains. # denotes activity against 119 viruses (Seaman panel); & denotes activity against 208 viruses (VRC panel). Bottom: Previously published rhesus and human V2 apex broadly neutralizing antibodies are included below the gray row for comparison; human antibodies are denoted with *. IC_50_ data for these antibodies are shown in italics when obtained from their respective publications. 119 virus panel data (#) for CH01, VRC26.08, and PCT64-35M were derived from CATNAP (https://www.hiv.lanl.gov/components/sequence/HIV/neutralization/). All large panel neutralization assays were performed in duplicate. **(d)** SHM versus antibody breadth on a 119-isolate panel is shown for representative antibodies, with V2 apex rhesus antibodies in red. **(e)** SHM versus antibody category is shown for antibodies with over 30% breadth on a 208-strain HIV-1 panel. V2 apex antibodies have SHM levels lower than other categories, though similar to those of the MPER category. Notably, V033-a.01 with 37% breadth showed substantially lower SHM.

**Figure S1.**
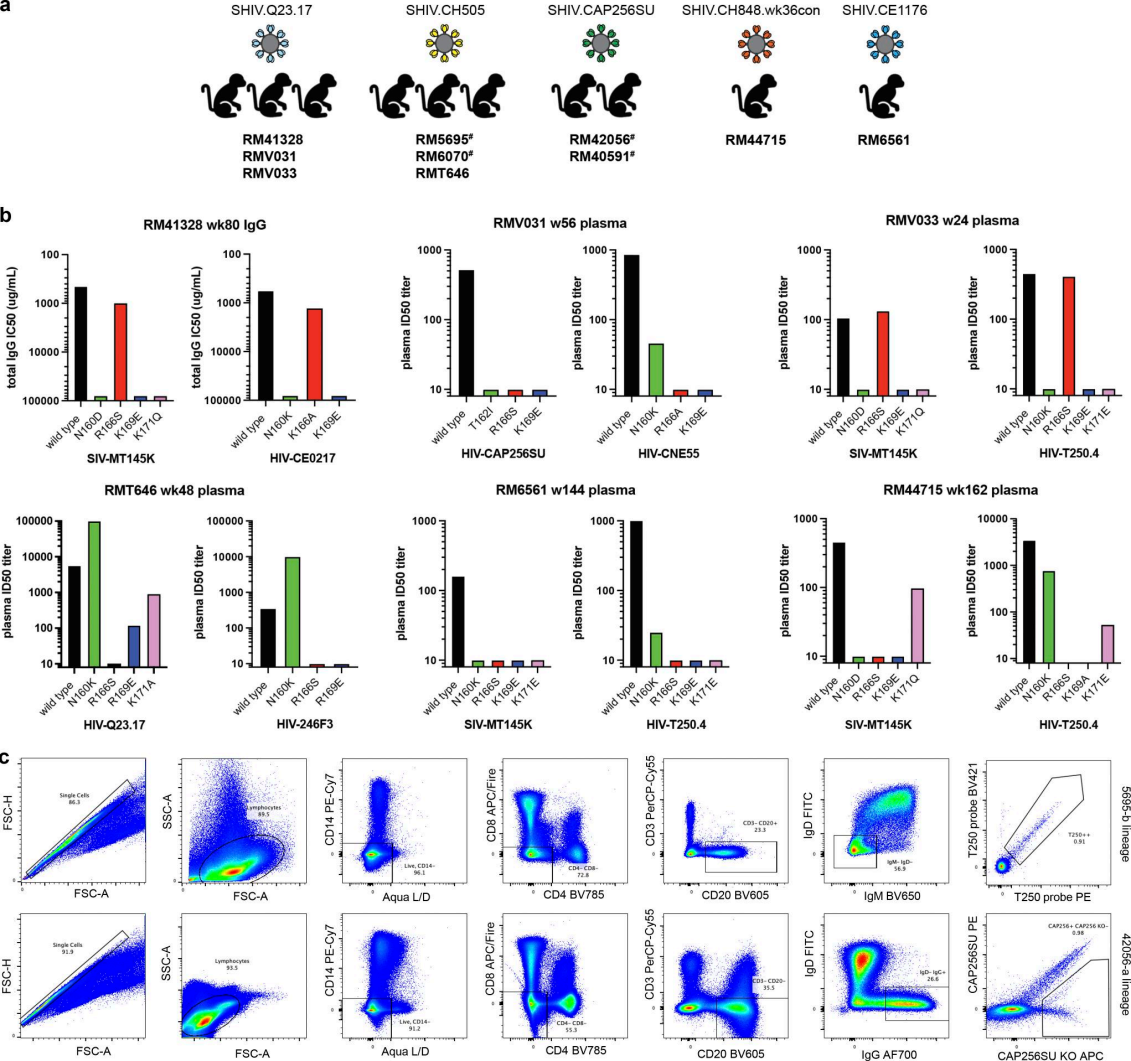
**Identification of V2 apex broadly neutralizing antibodies. (a)** Rhesus macaques from which V2 apex lineages have been isolated in this study are grouped by their respective infecting SHIV strain. Animals previously described by Roark et al. (2021) for polyclonal V2 apex mutational mapping are denoted with #. **(b)** Neutralization of two sets of heterologous wild-type and V2 apex epitope mutant viruses by rhesus macaque plasma or purified polyclonal IgG (RM41328) for SHIV-infected rhesus macaques reported in this study. Plasma neutralization assays were repeated twice. **(c)** Representative FACS gating schemes for the two different single-cell sorting strategies. Top: Identification of 5695-b lineage members by collecting double-positive cells stained with a heterologous Env SOSIP probe conjugated with two different fluorophores. Bottom: Identification of 42056-a lineage members by collecting single-positive cells staining with heterologous wild-type and C-strand mutant Env SOSIP probe pairs.

To characterize the molecular repertoire of rhesus V2 apex recognition, we used antigen-specific single-cell sorting to isolate mAbs from ten SHIV-infected rhesus macaques with polyclonal V2 apex–directed broadly neutralizing responses (Fig. 1 a, Fig. S1, and Table S1); nine of these were newly screened, and we also further screened B cells from macaque RM5695, from which we previously isolated RHA1. Memory B cells were sorted from peripheral blood mononuclear cells of each of the 10 selected rhesus macaque from a single time point using heterologous or epitope-specific Env SOSIP-probe pairs (Fig. S1 c and Table S2). Pairings comprised (1) a heterologous Env with different fluorophores, (2) two different heterologous Envs, or (3) an Env paired with a V2 apex–mutant trimer. By using PCR to amplify the paired immunoglobulin variable genes from single-cell transcripts (Wiehe et al., 2014; Mason et al., 2016), we recovered mAbs that belonged to individual expanded lineages from each rhesus macaque, with two distinct lineages from RM42056 and a second lineage from RM5695, each bearing atypically long ≥21 residue HCDR3s (Kabat numbering) with an overall electronegative charge due to an enrichment of anionic residues (Glu [E] and Asp [D]) (antibodies named to indicate rhesus ID-lineage.clone with a representative mAb from each lineage included in Fig. 1) (Fig. 1 b and Table S2). The HCDR3 features of the rhesus antibodies shown in Fig. 1 b were characteristic of human and rhesus broadly neutralizing antibodies that target the HIV-1 V2 apex site of vulnerability and must penetrate apical glycans to reach the shielded cationic C-strand (Mclellan et al., 2011; Andrabi et al., 2015; Gorman et al., 2016; Moore et al., 2017).

To assign accurately the germline genes and levels of SHM for each lineage, we performed next-generation sequencing of naïve B cell transcripts of each rhesus macaque and analyzed these sequences with IgDiscover (Corcoran et al., 2016) to curate personalized immunoglobulin gene libraries (Table S3). The heavy and light chain variable regions of each rhesus lineage were derived from unique recombined genetic origins, although the 42056-a and 42056-b lineages utilized the same germline VH4-NL_17*01_S2469 gene, and the T646-a and 41328-a lineages utilized similar alleles of the VL1-ABN gene (Fig. 1 b,Tables S2, and S3). SHM levels within all lineages were modest, with nucleotide divergence from each respective germline V gene ranging from 2–16% in heavy chain and 3–10% in light chain across all recovered antibodies. Only 3 of the 11 lineages contained insertions or deletions (indels) compared with germline: a single-residue deletion within HCDR2 of the 44715-a lineage, a two-residue insertion in the HCDR3 of the V031-a lineage, and a single-residue insertion within HCDR3 of the 6561-a.01 lineage. These SHM features are consistent with previously described human and rhesus V2 apex–directed broadly neutralizing antibodies, which typically require less affinity maturation and few or no indels to achieve breadth (Walker et al., 2009; Walker et al., 2011; Bonsignori et al., 2011; Doria-Rose et al., 2014; Landais et al., 2017) compared with human broadly neutralizing antibodies targeting other sites of vulnerability (Burton and Hangartner, 2016; Moore and Williamson, 2016; Kwong and Mascola, 2018; Griffith and Mccoy, 2021).

**Figure S2.**
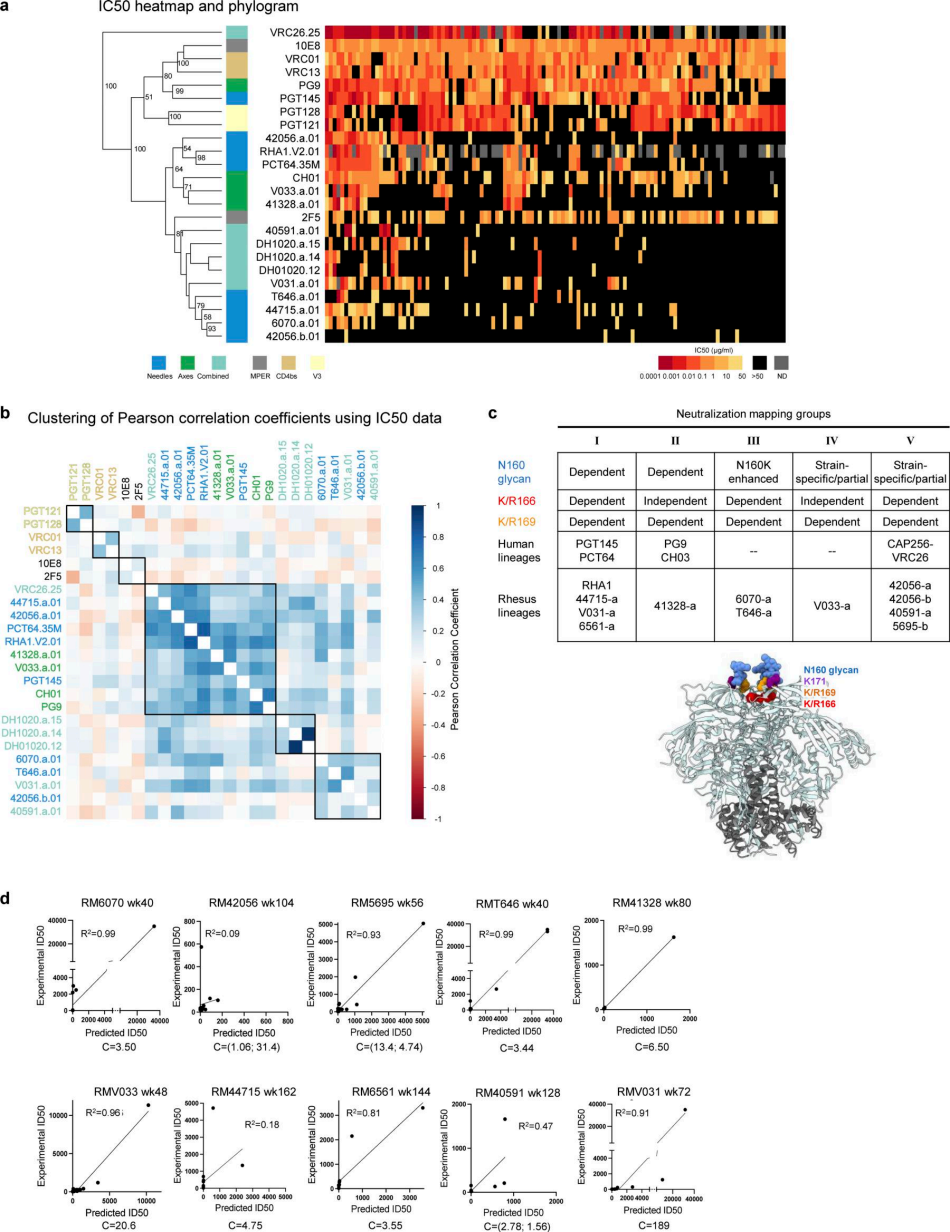
**Phenotypic analysis of isolated broadly neutralizing antibodies. (a)** Heatmap and phylogram based on hierarchical clustering of (log10-transformed) IC_50_ neutralization titers against a 119-heterologous virus panel. Epitope classes are shown next to the phylogram, and branch splits with >50% bootstrap support are indicated. **(b)** Hierarchical clustering of Pearson correlations of (log10-transformed) IC_50_ titers for the same pseudoviruses compared across broadly neutralizing antibodies. Of the seven clusters identified, three included V2 apex lineages. All axes, the majority of needles, and the only combined VRC26 clustered in the central cluster, while the remaining needles with two combined lineages formed the cluster at the bottom. DH1020 lineage members formed their own cluster. **(c)** Potent rhesus and human V2 apex**–**targeted lineages can be divided into five neutralization groups (I**–**V) based on mutant virus epitope mapping. Groups III and IV have not been previously described. The envelope trimer (PDB ID 4ZMJ) highlights the location of *N*-linked glycan and protein residue substitutions used for V2 apex mapping. Neutralization data values from mapping experiments are provided in Table S1. **(d)** Correlations between plasma neutralization ID_50_s and predicted neutralization by isolated mAbs at the specific concentration, “C,” which is provided in µg/ml. Neutralization data values for plasma and antibodies are provided in Table S1.

We then synthesized and expressed antibodies from each lineage and tested them for neutralization against a panel of 19 tier-2 neutralization-resistant viruses (Fig. 1 c and Table S1). The lineages exhibited a range of activity, with the broadest members of each lineage neutralizing 11–78% of the 19 heterologous viruses with a median IC_50_ ranging from 0.04 to 8.7 µg/ml (Fig. 1 c). These lineages were subsequently tested for neutralization against 119- and 208-strain panels of diverse HIV-1 strains and found to exhibit breadth of 4–37% with geomean IC_50_ of 0.28–19.8 µg/ml (Fig. 1 c and Table S1). Notably, these rhesus antibodies only partially segregated with known human V2 apex antibodies in an IC_50_ phylogram (Fig. S2 a); hierarchical clustering of Pearson correlations of (log10-transformed) IC_50_ titers, however, identified seven clusters, three of which were specific to V2 apex antibodies (Fig. S2 b). All axes, a majority of needles, and antibody CAP256-VRC26 clustered in a central subgroup; the remaining needles with two combined lineages formed another subgroup, and DH1020 lineage members formed their own subgroup.

To map phenotypically the epitope specificity of the newly identified lineages, we tested representative lineage members for neutralization against heterologous viruses bearing mutations at canonical V2 apex epitope residues 160, 166, 169, and 171 (Table S4). Removal of the N160 glycan or substitutions of positively charged residues abrogated or substantially reduced neutralization of these mutant heterologous viruses. Based on patterns of neutralization loss against viruses containing different canonical V2 apex mutations, we could divide the rhesus antibody lineages into five distinct phenotypic groups (Fig. S2 c): Three of these groups shared similar patterns with the three prototypic modes of HCDR3-dominated human V2 apex broadly neutralizing antibodies (Doria-Rose et al., 2014; Andrabi et al., 2015), while a fourth and fifth group exhibited novel phenotypes. The fourth group, comprising lineages 6070-a and T646-a, was distinguished by dramatic enhancement in neutralization potency (100- to 10,000-fold) against heterologous viruses lacking N160 glycan; this stands in contrast to previously reported V2 apex broadly neutralizing antibodies, which generally are N160 dependent. The fifth group included the V033-a lineage, which exhibited variable strain-specific dependence on N160 glycan for neutralization (Table S4), like the human antibody VRC26.25 (Doria-Rose et al., 2016). However, unlike VRC26.25, the V033-a lineage was not affected by mutations at residue 166 within the apex hole. Altogether, characterization of the 11 newly identified V2 apex–targeted neutralizing lineages from SHIV-infected rhesus macaques revealed antibodies that shared many immunogenetic and phenotypic features with previously described human V2 apex broadly neutralizing antibodies, though segregating into five distinct phenotypic groups based on their sensitivity to specific V2 apex substitutions in sites of paratope–epitope interaction (Fig. S2 c).

### V2 apex antibodies with low SHM relative to breadth

V2 apex–directed antibodies generally have less affinity maturation than some of the other categories of HIV-1 broadly neutralizing antibodies, such as those targeting the CD4-binding site, with longitudinal analyses indicating that cross-clade neutralization can be achieved rapidly, in some cases within a few weeks or months after initial B cell activation (Walker et al., 2010; Landais and Moore, 2018). We compared the new V2-identified antibodies against all antibodies in the CATNAP (Yoon et al., 2015) database with 119-strain data and at least 5% cross-clade neutralization breadth, observing the newly identified antibodies to be notable for their relatively low SHM relative to neutralization breadth (Fig. 1 d).

V033-a lineage was notable because it was isolated just 24 wk after SHIV infection, exhibited particularly low levels of SHM (2.0–6.8% VH nucleotide), with antibody V033-a.01 (<5% SHM nucleotide; <10% SHM amino acid) neutralizing 31% of a 119-strain panel and 37% of a 208-strain panels with geomean IC_50_s of 0.45 and 0.60 µg/ml, respectively). The level of SHM for V033-a.01 was substantially lower than previously characterized antibodies of at least 30% breadth on the panel of 208-HIV-1 strains (Fig. 1 e).

Overall, V2 apex antibodies trended to lower SHM relative to neutralization breadth, which was particularly notable with antibody V033-a.01.

### Correlations between isolated antibody IC_50_s and plasma ID_50_s reveal a single V2 apex broadly neutralizing lineage to account for neutralization breadth in most macaques

We assessed the degree to which the isolated antibodies could recapitulate plasma neutralization. A single mAb in 5 of the 10 analyzed macaques was able to recapitulate most of the heterologous neutralization (ID_50_) in each respective macaque plasma, with correlations ranging from 0.91 to 0.99 for the 5 macaques with good recapitulation (Fig. S2 d and Table S4). Two other macaques, RM6561 and RM40591, had moderate recapitulation with R^2^ correlation of 0.81 and 0.47, respectively. For RM40591, the best recapitulation occurred with two antibody clones from the 40591-a lineage.

Three macaques did not show V2 apex lineage recapitulation of neutralization breadth. First was RM5695, from which we isolated both antibodies RHA1 (Roark et al., 2021) and 5695-b; while RHA1 largely recapitulated the animal’s plasma breadth, the 5695-b lineage did contribute to neutralization breadth with a combined antibody R^2^ correlation of 0.93. Second was animal RM6561 (R^2^ correlation for 6561-a.01 of 0.81); a second broadly neutralizing lineage targeting the fusion peptide was also isolated from this animal (G.M. Shaw, personal communication). Third, RM44715 (R^2^ correlation for 44715-a.01 of 0.18) also harbored a second neutralizing antibody lineage that targeted the V3-glycan supersite (G.M. Shaw, personal communication). Thus, except for three macaques in which two lineages appeared responsible for breadth, in most of the macaques, a single lineage could account for observed cross-clade neutralization breadth.

### Rhesus V2 apex–targeted lineages all utilize the same DH3-15*01 gene

**Figure 2.**
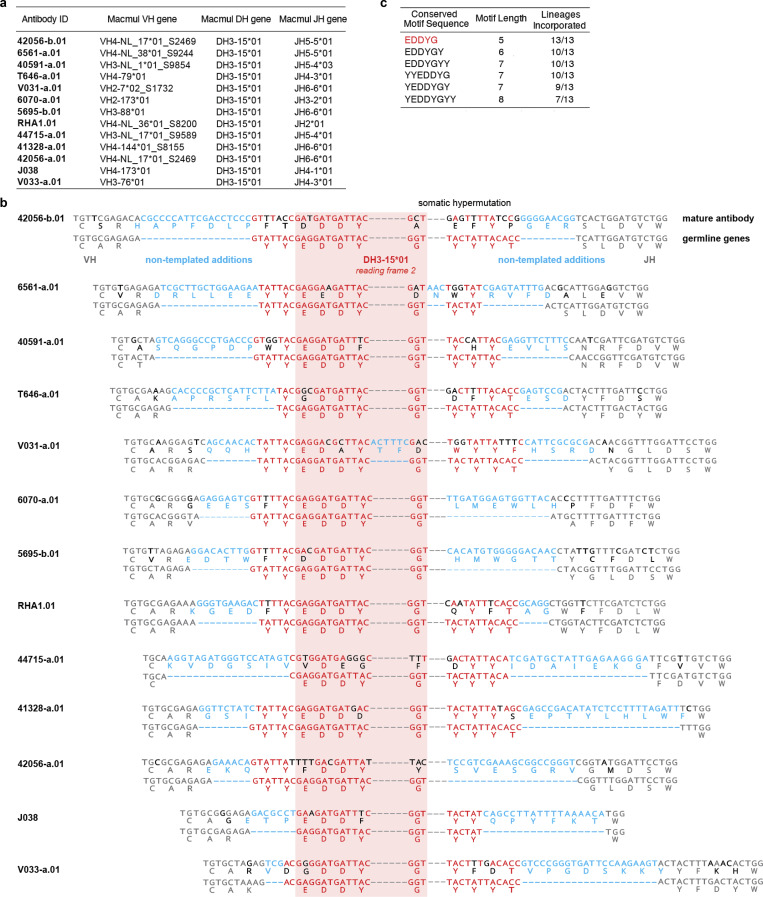
All SHIV-induced V2 apex–directed lineages are derived from the same rhesus DH3-15*01 gene in reading frame two and invariantly acquire a minimal five-residue motif. (a) Germline VH, DH, and JH genes for all potently neutralizing rhesus V2 apex**–**directed lineages described here and previously (13 total). **(b)** VDJ junction analysis of a representative antibody from each rhesus lineage. The respective germline VH, DH, and JH gene sequences are truncated and aligned to each VDJ junction, and each VDJ junction is aligned with respect to the DH gene. VH and JH nucleotides and residues are colored gray, non-templated nucleotides and residues (insertions and N/P additions) are colored blue, DH-gene nucleotides and residues are colored red, and SHM is colored black; we do not interpret SHM within non-templated regions. The reading frame in which each DH gene has been incorporated is denoted. The five-residue DH3-15*01 motif (EDDYG) acquired by all 13 lineages during VDJ recombination is highlighted with transparent red shading. **(c)** List of conserved DH3-15*01 residue motifs of varying length that are acquired by at least half of the rhesus lineages during VDJ recombination. The five-residue EDDYG motif described in panel b is written in red.

Our initial immunogenetic analysis revealed that each of the 11 newly identified rhesus lineages were derived from unique heavy and light chain V gene and J gene pairs, but all utilized the same DH3-15*01 gene (Ramesh et al., 2017) (alternate designation: DH3-9*01 [Vázquez Bernat et al., 2021]) (Fig. 2 a and Table S2). Moreover, previously reported rhesus V2 apex broadly neutralizing antibody RHA1 also utilized DH3-15*01 (Roark et al., 2021) (we note that the V2 apex neutralizer, J038, which binds with a three-antibody per trimer stoichiometry, also used DH3-15*01 [Gao et al., 2022]). We confirmed the presence of the exact germline DH3-15*01 sequence in each of the 10 rhesus macaques for which we had naïve B cell transcript sequences (Table S3). To gain further insight into the utilization of this D gene, we performed VDJ junctional analysis (residues C92_H_ to W103_H_; Kabat numbering) for all 11 rhesus V2 apex lineages along with the two previously reported lineages. To facilitate and visualize comparisons, we aligned the 13 sets of germline and VDJ junction sequences against DH3-15*01 (Fig. 2 b).

**Figure S3.**
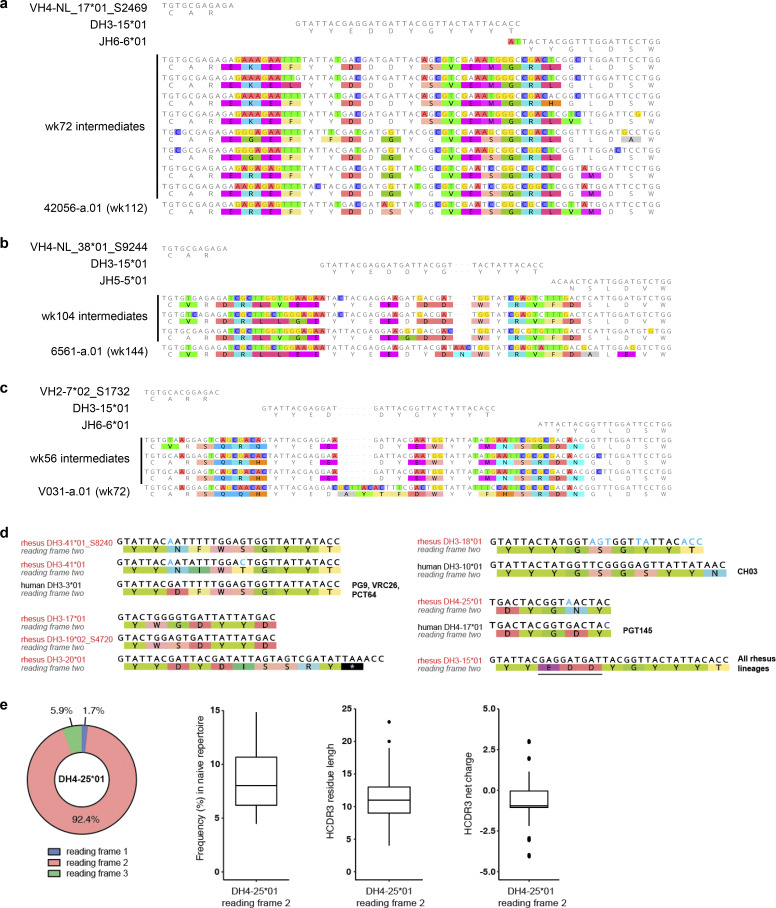
**Rhesus macaque and human immunoglobulin sequence analysis. (a–c)** Ancestral clonal sequences identify HCDR3 indels and VDJ gene contributions and confirm the acquisition of a five-residue EDDYG motif for rhesus lineages with significant somatic mutation within D gene segments. HCDR3 alignments of the rhesus antibodies (a) 42056-a.01, (b) 6561-a.01, and (c) V031-a.01 to their respective VDJ germline genes and ancestral lineage intermediate sequences identified from peripheral memory B cells at the indicated time points. Mismatches to the germline V, D, and J genes are highlighted. **(d)** Human V2 apex broadly neutralizing lineage D genes are aligned with their rhesus D gene homologs. Nucleotide differences in rhesus homologs are colored in blue. Non-homolog rhesus D genes encoding the “YYD” motif in human DH3-3*01 are included below that alignment. Rhesus DH3-15*01 is included for reference with the unique three-residue anionic motif (EDD) underlined. All rhesus sequences are labeled in red. **(e)** Left, proportion of reading frame usage among naïve rhesus B cells derived from DH4-25*01, the rhesus homolog of the human PGT145 lineage DH4-17*01 gene, in the peripheral Indian rhesus macaque repertoire. Right, box plots showing in order: the frequency of DH4-17*01 usage in reading frame two among all naïve B cells in the peripheral rhesus repertoire; HCDR3 length distribution of naïve rhesus B cells in the peripheral rhesus repertoire derived from DH4-17*01 in reading frame two; HCDR3 net charge distribution of naïve rhesus B cells in the peripheral repertoire derived from DH4-17*01 in reading frame two. Charge calculations only consider amino acid residues and not predicted sites of tyrosine sulfation.

Strikingly, DH3-15*01 was invariantly incorporated in reading frame two by each lineage. In addition to being rich in anionic residues, rhesus and human V2 apex broadly neutralizing antibody HCDR3s also contain many aromatic residues (most commonly Tyr [Y]) (Fig. 1 b and Table S2) (Walker et al., 2009; Walker et al., 2011; Bonsignori et al., 2011; Doria-Rose et al., 2014; Roark et al., 2021). A majority of these characteristic residues in rhesus lineages were contributed by DH3-15*01, which could only be achieved by translation in the second reading frame. The DH3-15*01 start positions (the first residue fully coded by D gene nucleotides) for all rhesus lineages spanned just five residues from 97_H_ to 100b_H_, with the most common positions, 98_H_ and 99_H_, shared by three lineages each. DH3-15*01 added significantly to the atypical length of each HCDR3 by contributing 21 to 33 D gene nucleotides, resulting in a minimal germline 15 nucleotide sequence that was incorporated into all 13 rhesus lineages (highlighted by red shading in the alignment) (Fig. 2 b). This conserved D gene sequence yielded a five-residue EDDYG motif that was shared by each rhesus lineage inferred unmutated common ancestor following VDJ recombination (Fig. 2 c). This motif was not commonly subjected to SHM, as most lineages (8 of 13) had zero to one mutated motif residue in mature clonal sequences. Even longer seven-residue motifs, YYEDDYG or EDDYGYY, achieved by including two consecutive germline-encoded Tyr residues at either the N or C termini, were observed in 9 of 13 lineages (Fig. 2, b and c). Identification of 6561-a and V031-a lineage sequences from time points preceding mAb isolation confirmed an insertion of one and two residues, respectively, within the original D gene fragment during lineage development (Fig. S3, a–c).

Overall, this analysis indicates DH3-15*01 gene usage in reading frame two to be a signature feature of HCDR3 ontogenies in rhesus V2 apex broadly neutralizing lineages. The invariant incorporation of the EDDYG motif during each VDJ recombination event in otherwise genetically diverse templated and non-templated backgrounds suggests this sequence to be important for rhesus antibody recognition of the HIV-1 V2 apex.

### Cryo-EM structures of rhesus antibody lineages reveal similarity to canonical human modes of V2 apex recognition

To provide molecular characterization of V2 apex recognition and the specific role of the DH3-15*01 gene, we determined the structures of Fab’s from nine of the new rhesus lineages in complex with prefusion-stabilized HIV-1 Envs using single-particle cryo-EM (Data S1 and Table S5). Structural analysis revealed that these rhesus lineages recapitulated canonical human modes of apex insertion and C-strand hydrogen bonding (or their combination) (Andrabi et al., 2015; Gorman et al., 2016).

#### Inserting needle-like recognition

**Figure 3.**
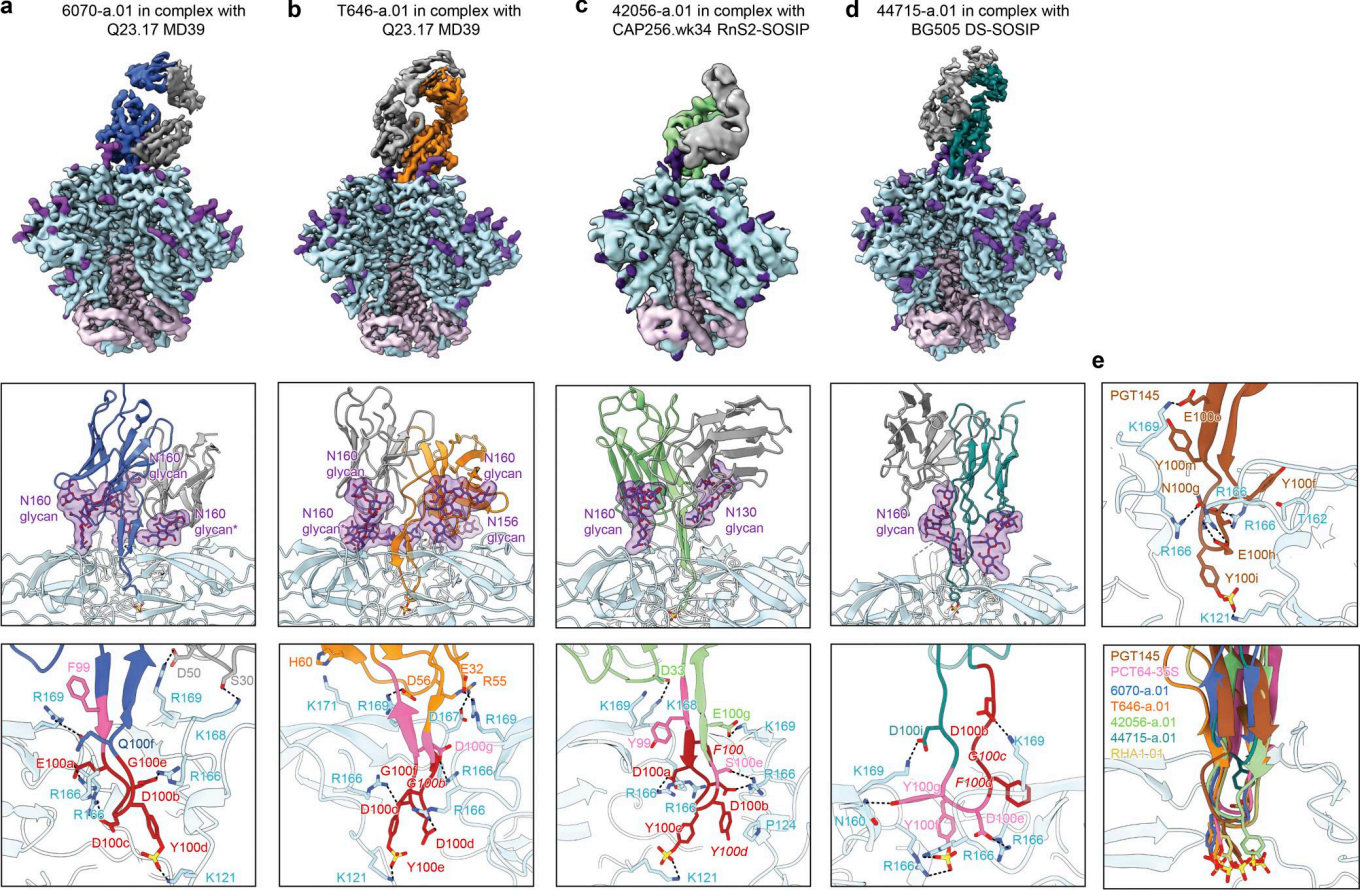
**Cryo-EM structures reveal needle-like modes of V2 apex recognition to be a reproducible antibody extended class in rhesus macaques. (a)** Top: Cryo-EM reconstruction of 6070-a.01 in complex with Q23.17 MD39 Env at 3.6-Å resolution. The 6070-a.01 heavy and light chains are colored blue and gray, respectively. Envelope gp120, gp41, and *N*-linked glycans are colored turquoise, pink, and purple, respectively. Middle: Expanded interface view of 6070-a.01 from the top panel to highlight binding position and interactions with apical envelope glycans. Glycans bound by 6070-a.01 are shown in stick representation with transparent surfaces. The N160 glycan reoriented outward and away from the threefold trimer axis is denoted with *. Sulfated tyrosine residues are shown in stick representation to highlight their position within the trimer. Bottom: Further expanded interface view of 6070-a.01 to highlight interactions with apical envelope residues. Interacting residues are depicted in stick representation. Residues at positions corresponding to the conserved five-residue DH3-15*01 gene motif are colored dark red, while the remaining D gene residues are colored pink. Conserved motif position labels are italicized when subjected to SHM. Nitrogen atoms are colored blue, oxygen atoms are colored bright red, and sulfur atoms are colored yellow. Hydrogen bonds and salt bridges (distance < 3.3 Å) are depicted with dashed lines. **(b)** Top: Cryo-EM reconstruction of T646-a.01 in complex with Q23.17 MD39 Env at 3.5-Å resolution. The T646-a.01 heavy chain is colored orange, and the remainder of the complex is colored similarly to panel a. Middle: Expanded interface view of T646-a.01 from the top panel to highlight binding position and apical glycan interactions is shown similarly to panel a, including the N160 glycan reoriented into a horizontal conformation denoted with *. Bottom: Further expanded interface view of T646-a.01 to highlight apical residue interactions is shown similarly to panel a. **(c)** Top: Cryo-EM reconstruction of 42056-a.01 in complex with CAP256.wk34.c80 RnS2 SOSIP determined at 4.1-Å resolution. The 42056-a.01 heavy chain is colored light green, and the remainder of the complex is colored similarly to panel a. Middle: Expanded interface view of 42056-a.01 from the top panel to highlight binding position and apical glycan interactions is shown similarly to panel a. Bottom: Further expanded interface view of 42056-a.01 highlight apical residue interactions is shown similarly to panel a. **(d)** Top: Cryo-EM reconstruction of 44715-a.01 in complex with BG505 DS-SOSIP at 3.9-Å resolution. The 44715-a.01 heavy chain is colored teal, and the remainder of the complex is colored similarly to panel a. Middle: Expanded interface view of 44715-a.01 from the top panel to highlight binding position and apical glycan interactions is shown similarly to panel a. Bottom: Further expanded interface view of 44715-a.01 to highlight apical residue interactions is shown similarly to panel a. **(e)** Top: Expanded HCDR3 interface view of PGT145 (PDB ID 5V8L) to highlight apical residue interactions is shown similarly to panel a. The tyrosine sulfation posttranslational modification of Y100i was not included in this structure and therefore modeled here. Bottom: Expanded HCDR3 interface side view of the alignment of envelope complex structures of 6070-a.01, T646-a.01, 42056-a.01, and 44715-a.01 determined here to envelope complexes with human Fab’s PCT64-35S (PDB ID 7T74) and PGT145 (PDB ID 5V8L) and rhesus Fab RHA1.V2.01 (PDB ID 6XRT). Alignments were made with gp120 from each complex. Only gp120 of the 6070-a.01 complex is shown for clarity. Sulfated tyrosine residues are shown to highlight their positioning within the trimer.

The cryo-EM structures of 6070-a.01, T646-a.01, 42056-a.01, and 44715-a.01 (solved with 3D molecular reconstructions at 3.6-Å, 3.5-Å, 4.2-Å, and 3.9-Å resolution, respectively) revealed modes of recognition similar to antibodies PGT145 and PCT64, which represent an extended human broadly neutralizing antibody class that utilizes a needle-like hole-insertion mechanism to recognize the V2 apex (Fig. 3 e) (Walker et al., 2011; Landais et al., 2017; Lee et al., 2017; Rantalainen et al., 2018). A single Fab of each of these antibodies bound at the Env C3 symmetry axis with extended HCDR3s inserted directly into the cationic trimer hole (Fig. 3, a–d). Each lineage recognized two or more apical glycans from multiple protomers, resulting in 41–55% of their respective interactive surface areas being contributed by glycan interfaces (Table S6). This was comparable to the glycan fraction of interactive surfaces for PGT145 (45%) and PCT64-35s (56%). 6070-a.01 and T646-a.01 each recognized N160 glycan from all three protomers despite comprising the phenotypic neutralization group enhanced by N160 glycan removal (Fig. S2 c and Table S4). However, both lineages reoriented one of these glycans outward from the trimer C3 axis (6070-a) or into a horizontal conformation parallel with the Fab-combining surface (T646-a) (denoted with * in Fig. 3, a and b); this is in contrast to other human and rhesus PGT145-like antibodies that accommodate N160 glycans in a more vertical conformation similar to their conformation on the unliganded trimer. The induced glycan reorientation to accommodate 6070-a.01 and T646-a.01 is likely a barrier to binding, resulting in the enhanced potency of these antibodies once N160 glycan has been removed.

The four new lineages each interacted with conserved cationic amino acids from all three protomers lining the trimer apex hole, most commonly through electrostatic interactions with Env residues 166 and 169. The 6070-a.01 complex revealed a three-residue anionic motif (E100a_HCDR3_, D100b_HCDR3_, and D100c_HCDR3_) to form salt bridges with all three R166 residues, while F100 and Q100f_HCDR3_ together interacted with R169 from a single protomer, and light chain residues S30_LCDR1_ and D50_LCDR2_ engaged K168 and R169 from a second protomer (Fig. 3 a, bottom). T646-a.01 similarly formed salt bridges with R166 from all three protomers mediated by HCDR3 residues D100c, D100d, and D100g, while R169 from two different protomers was recognized by E32_HCDR1_ and D56_HCDR2_ (Fig. 3 b, bottom). For the 42056-a.01 complex, consecutive anionic residues D100a_HCDR3_ and D100b_HCDR3_ formed salt bridges with R166 from two protomers, while the third R166 residue was engaged by cation–π interactions with F100_HCDR3_ (Fig. 3 c, bottom). In addition, K168 and K169 from two different protomers were recognized through salt bridges formed with D33_LCDR1_ and E100g_HCDR3_, respectively. The 44715-a.01 paratope recognizing Env was entirely comprised of HCDR3, which included F100d_HCDR3_ and Y100g_HCDR3_ stabilizing the elongated aliphatic chains of K169 on two protomers, and salt bridges formed between cationic residues on all three protomers: D100a_HCDR3_ interacted with K169, D100i_HCDR3_ with another K169, and D100e_HCDR3_ with R166 (Fig. 3 d, bottom). The penetrance of these needle-like HCDR3s into the trimer hole to recognize Env residues 166 and 169 was consistent with mutations at these positions to confer complete loss of neutralization sensitivity (Fig. S2 c and Table S4).

**Figure S4.**
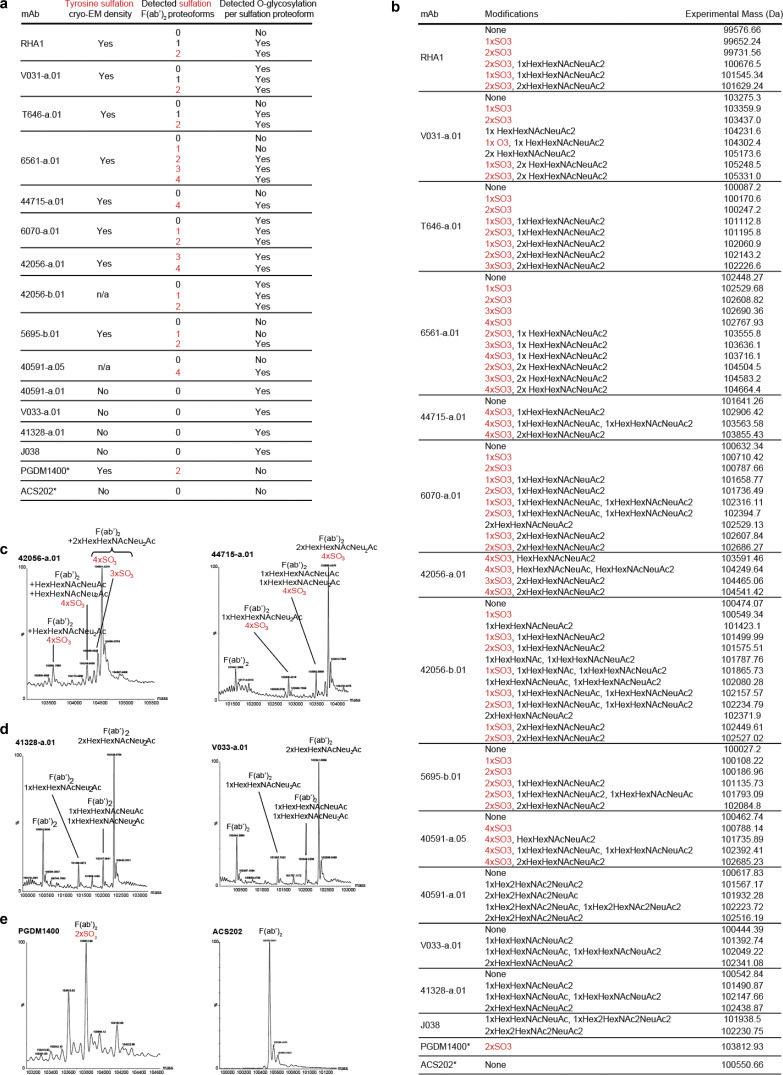
**Rhesus V2 apex lineages bear tyrosine sulfation and *O*-linked glycosylation posttranslational modifications. (a)** Summary of posttranslational modifications detected on F(ab’)2-digested rhesus and human antibodies by mass spectroscopy. Human antibodies are denoted with *. The number of sulfation groups detected per proteoform is written in red. All rhesus antibodies contained various types of *O*-linked glycosylation, while human antibody controls did not. Antibodies without structural data are denoted with n/a (not applicable). **(b)** Full list of individual detected experimental masses and corresponding deconvoluted posttranslational modifications that were summarized in panel (a). The number of sulfation groups detected per modification is written in red (#xSO3). **(c)** Representative deconvoluted mass spectra for rhesus lineage F(ab’)2 subunits with tyrosine sulfation peaks. Y axis: relative intensity. X axis: mass (Da). **(d)** Representative deconvoluted mass spectra for rhesus lineage F(ab’)2 subunits without tyrosine sulfation peaks. Y axis: relative intensity. X axis: mass (Da). **(e)** Deconvoluted mass spectra for human F(ab’)2-digested antibodies PGDM1400 and ACS202, which serve as positive and negative controls for tyrosine sulfation, respectively. Y axis: relative intensity. X axis: mass (Da).

In addition, similar to antibody PGT145, antibodies 6070-a.01, T646-a.01, and 42056-a.01 each contained tyrosine-sulfated HCDR3 tips that penetrated deep enough into the trimer to form salt bridges with conserved residue K121 (Fig. 3, a–c; and Fig. S4). 44715-a.01 also had a tyrosine-sulfated HCDR3 tip, but it did not insert as deeply into the trimer; instead, this sulfated tyrosine formed salt bridges with R166 from two different protomers (Fig. 3 d and Fig. S4). An overlay of these four structures with the Env complexes of PGT145, PCT64-35S, and RHA1 revealed an alignment of HCDR3 loops extending along the C3 axis into the trimer despite a constellation of unique Fab orientations (Fig. 3 e and Data S1-Fig. 10 a). Apart from 44715-a.01, whose HCDR3 penetrated ∼10-Å shallower than the other lineages, the sulfated HCDR3 tips of the other six lineages aligned within the middle of the trimer. In particular, the sulfated tyrosine residues in five of these structures were positioned at precisely the same location, while this sulfated tyrosine of the sixth structure (42056-a.01) was just one residue position downstream.

Together, these data demonstrate chemical and structural mimicry of the human PGT145 lineage to be a reproducible mode of broadly neutralizing V2 apex recognition in rhesus macaques.

#### Axe-like recognition: C-strand hydrogen bonding

**Figure 4.**
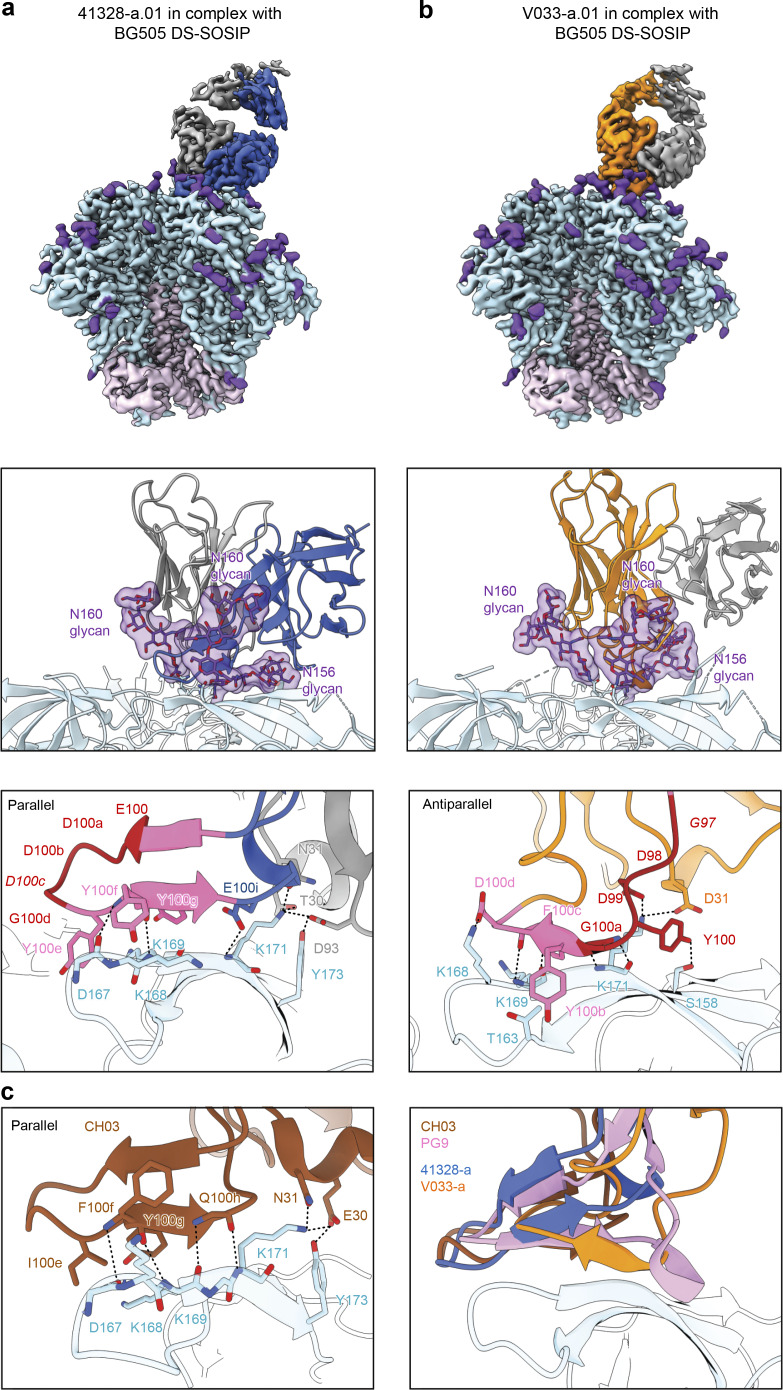
**Cryo-EM structures reveal axe-like modes of V2 apex recognition to be a reproducible antibody extended class in rhesus macaques. (a)** Top: Cryo-EM reconstruction of 41328-a.01 in complex with BG505 DS-SOSIP at 2.9-Å resolution. The 41328-a.01 heavy and light chains are colored blue and gray, respectively. Envelope gp120, gp41, and *N*-linked glycans are colored turquoise, pink, and purple, respectively. Middle: Expanded interface view of 41328-a.01 from the top panel to highlight binding position and interactions with apical envelope glycans. Glycans bound by 41328-a.01 are shown in stick representation with transparent surfaces. Bottom: Further expanded interface view of 41328-a.01 to highlight interactions with apical envelope residues. Interacting residues are depicted in stick representation. Residues at positions corresponding to the conserved five-residue DH3-15*01 gene motif are colored dark red, while the remaining D gene residues are colored pink. Conserved motif position labels are italicized when subjected to SHM. Nitrogen atoms are colored blue, and oxygen atoms are colored bright red. Hydrogen bonds and salt bridges (distance < 3.3 Å) are depicted with dashed lines. The orientations of the C-strand and HCDR3 β-strand mainchain interactions are labeled in the top left corner. **(b)** Top: Cryo-EM reconstruction of V033-a.01 in complex with BG505 DS-SOSIP at 3.1-Å resolution. The V033-a.01 heavy chain is colored orange, and the remainder of the complex is colored similarly to panel a. Middle: Expanded interface view of V033-a.01 from the top panel to highlight binding position and apical glycan interactions is shown similarly to panel a. Bottom: Further expanded interface view of V033-a.01 to highlight apical residue interactions is shown similarly to panel a. **(c)** Right: Expanded HCDR3 interface view of CH03 (PDB ID 5ESV) to highlight apical residue interactions is shown similarly to panel A. Left: Expanded HCDR3 interface side view of the alignment of SOSIP complex structures of 41328-a.01 and V033-a.01 determined here to an envelope complex with human Fab PG9 (PDB ID 8FL1) and V1V2**–**scaffold complex with human Fab CH03 (PDB ID 5ESV). Alignments were made with the V1V2 region from each complex. Only gp120 of the 41328-a.01 complex is shown for clarity.

Cryo-EM structures of 41328-a.01 and V033-a.01 in complex with BG505 DS-SOSIP revealed modes of V2 apex recognition similar to human broadly neutralizing antibodies PG9 and CH03, which utilize β-strand pairing of the V2 C-strand as a focus of trimer apex recognition (Fig. 4 c) (Walker et al., 2009; Bonsignori et al., 2011; Mclellan et al., 2011; Gorman et al., 2016). While 41328-a.01 solely bound Env with a 1:1 stoichiometry that yielded a single 3D reconstruction of 2.9-Å resolution, we obtained reconstructions for 1, 2, and 3 V033-a.01 Fab-bound Env complexes (Data S1-Fig. 10 c). To facilitate comparison with other rhesus and human lineages, we solved the atomic structure of V033-a.01 using the 3D reconstruction of the single Fab-bound complex, which extended to 3.1-Å resolution. 41328-a.01 and V033-a.01 Fab’s each exhibited asymmetric recognition of the trimer apex by penetrating between the N156 and N160 glycans of one protomer and binding a second N160 glycan from an adjacent protomer (Fig. 4, a and b). These apical glycan interactions were substantial, contributing 52% and 68% of the total interactive surface areas for 41321-a.01 and V033-a.01, respectively (Table S6). The structures also revealed both lineage HCDR3s to contain an axe-like subdomain that recognized the C strand from a single protomer through parallel (41328-a.01) or antiparallel (V033-a.01) β-strand interactions. Two parallel hydrogen bonds formed between the mainchains of 41328-a.01 residue Y100f_HCDR3_ and Env residues 167 and 168, and four antiparallel hydrogen bonds formed between the mainchains of V033-a.01 residues F100c_HCDR3_ and G100a_HCDR3_ and Env residues 169 and 171. For 41328-a.01, a third mainchain hydrogen bond was formed between the backbone amide of Env residue 171 and the side chain of E100i_HCDR3_.

We also observed a number of interactions with Env residue side chains for both structures that closely resembled those of CH03 (Fig. 4 c, left). In the 41328-a.01 complex, a string of three aromatic residues (Y100e_HCDR3_, Y100f_HCDR3_, and Y100g_HCDR3_) stabilized the extended aliphatic chains of Env C-strand residues K168 and K169 in an identical manner to the string of three hydrophobic residues (I100e_HCDR3_, F100f_HCDR3_, and Y100g_HCDR3_) utilized by CH03. V033-a.01 similarly utilized two consecutive aromatic residues (Y100b_HCDR3_ and F100c_HCDR3_) to stabilize the aliphatic chains of K168 and K169, while also engaging K168 through a salt bridge mediated by D100d_HCDR3_. 41328-a.01 further engaged the C strand through light chain residues T30_LCDR1_ and N31_LCDR1_, forming hydrogen bonds with K171 and D93 _LCDR3_, forming a salt bridge with K171 and a hydrogen bond with Y173; these interactions with K171 and Y173 were strikingly similar to those of CH03 mediated by heavy chain residues E30_HCDR1_ and N31_HCDR1_. V033-a.01 could instead engage K171 with two potential salt bridges through D31_HCDR1_ and D99_HCDR3_. The ability of mutations at C-strand residues 169 and 171, but not residue 166, to confer neutralization resistance to the rhesus axe-like lineages is consistent with the lack of HCDR3 insertion into the trimer hole (Fig. S2 c and Table S4).

An overlay of the 41328-a.01 and V033-a.01 structures with the Env complex of PG9 and the V1V2 scaffold complex of CH03 revealed an approximate alignment of their respective HCDR3 subdomains positioned parallel with the trimer apex plane (Fig. 4 c, right). The four structures demonstrated Fab’s to engage Env with one of two different heavy and light chain orientations that were rotated by ∼90° and did not segregate by species (Data S1-Fig. 10 b). There was exceptional overlap between the HCDR3s of 41328-a.01 and CH03, while the longer HCDR3 of PG9 extended further back along the C strand before a helical turn redirected and closed the subdomain. Although the HCDR3 length of V033-a.01 was the same as 41328-a.01 and just one residue shorter than CH03, its unique antiparallel subdomain was significantly more compact and did not extend beyond the C strand toward the trimer C3 axis like the other PG9-like lineages. This smaller footprint provides a structural explanation for the ability of V033-a.01 to also exhibit 1:2 and 1:3 binding stoichiometries with the prefusion-closed conformation of Env (Data S1-Fig. 10 c).

Collectively, these structures demonstrate chemical and structural mimicry of the human PG9 and CH01 lineages and show β-strand pairing with the C strand at the trimer apex to be a reproducible mode of broadly neutralizing V2 apex recognition in rhesus macaques.

#### “Combined” mode recognition

**Figure 5.**
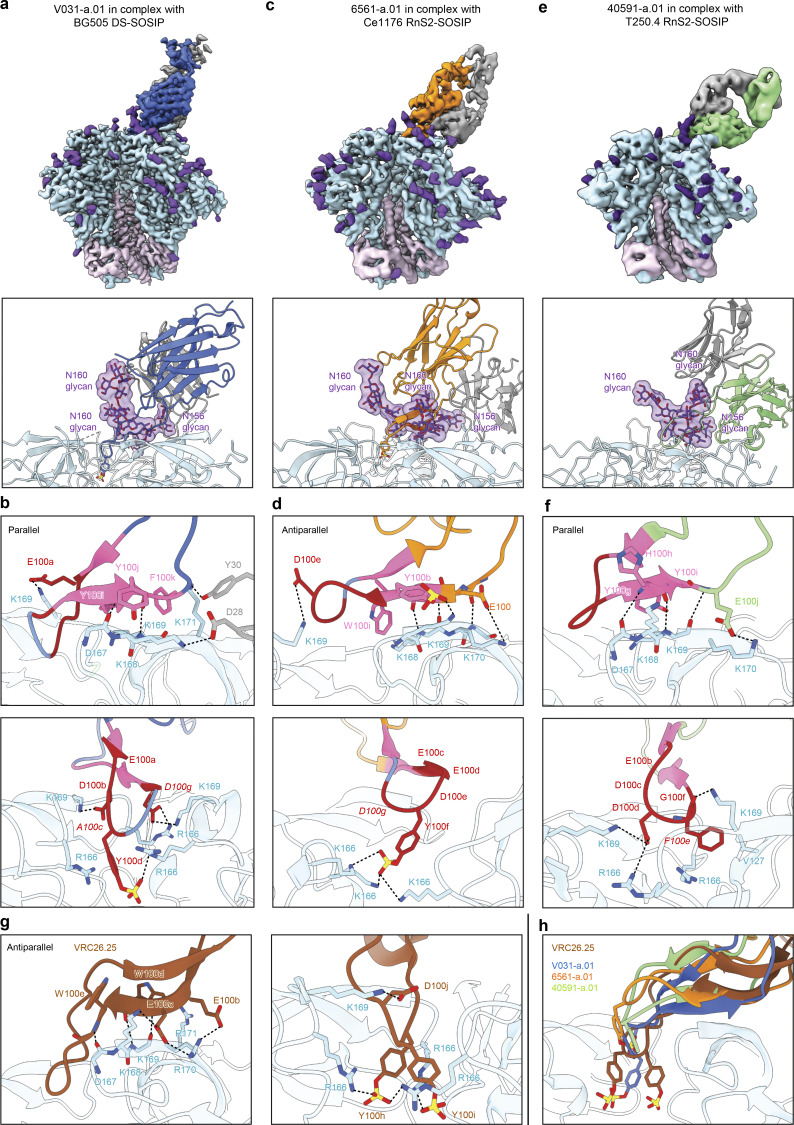
**Cryo-EM structures reveal combined modes of V2 apex recognition to be a reproducible antibody extended class in rhesus macaques. (a)** Top: Cryo-EM reconstruction of V031-a.01 in complex with BG505 DS-SOSIP at 3.1-Å resolution. The V031-a.01 heavy and light chains are colored blue and gray, respectively. Env gp120, gp41, and *N*-linked glycans are colored turquoise, pink, and purple, respectively. Bottom: Expanded interface view of V031-a.01 from the top panel to highlight binding position and interactions with apical envelope glycans. Glycans bound by V031-a.01 are shown in stick representation with transparent surfaces. Sulfated tyrosine residues are shown in stick representation to highlight their position within the trimer. **(b)** Further expanded interface views of V031-a.01 to highlight interactions with apical envelope residues. Interacting residues are depicted in stick representation. Residues at positions corresponding to the conserved five-residue DH3-15*01 gene motif are colored dark red, while the remaining D gene residues are colored pink. Conserved motif position labels are italicized when subjected to SHM. Nitrogen atoms are colored blue, oxygen atoms are colored bright red, and sulfur atoms are colored yellow. Hydrogen bonds and salt bridges (distance < 3.3 Å) are depicted with dashed lines. Top: interactions made with the primary recognized C strand. The orientations of the C-strand and HCDR3 β-strand mainchain interactions are labeled in the top left corner. Bottom: Interactions mediated by HCDR3 residues inserted into the trimer hole. **(c)** Top: Cryo-EM reconstruction of 6561-a.01 in complex with Ce1176 RnS2 SOSIP at 4.1-Å resolution. The 6561-a.01 heavy chain is colored orange, and the remainder of the complex is depicted similarly to panel a. Bottom: Expanded interface view of 6561-a.01 from the top panel to highlight binding position and interactions with apical glycans is shown similarly to panel a. **(d)** Further expanded interface views of 6561-a.01 to highlight interactions with apical residues are shown similarly to panel b. **(e)** Top: Cryo-EM reconstruction of 40591-a.01 in complex with T250.4 RnS2 SOSIP at 4.2-Å resolution. The 40591-a.01 heavy chain is colored orange, and the remainder of the complex is colored similarly to panel a. Bottom: Expanded interface view of 40591-a.01 from the top panel to highlight binding position and interactions with apical Env glycans is shown similarly to panel a. **(f)** Further expanded interface views of 40591-a.01 to highlight interactions with apical Env residues are shown similarly to panel b. **(g)** Expanded HCDR3 interface views of VRC26.25 (PDB ID 6VTT) to highlight apical envelope residue interactions are shown similarly to panel b. Left: Interactions are mediated by HCDR3 residues inserted into the trimer hole. Right: Interactions mediated by all other Fab residues. **(h)** Expanded HCDR3 interface side view of the alignment of envelope complex structures of V031-a.01, 6561-a.01, and 40591-a.01 determined here to the envelope complex of VRC26.25 (PDB ID 6VTT). Alignments were made with gp120 from each complexes, while only gp120 of the VRC26.25 complex is shown for clarity. Sulfated tyrosine residues are shown to highlight their positioning within the trimer. Residue F100e of 40591-a.01 is also shown since it is similarly inserted into the trimer hole but cannot be modified posttranslationally.

The cryo-EM structures of V031-a.01, 6561-a.01, and 40591-a.01 (determined with molecular reconstructions at 3.1-Å, 4.1-Å, and 4.2-Å resolution, respectively) revealed modes of V2 apex recognition similar to human broadly neutralizing antibody CAP256–VRC26.25 (Doria-Rose et al., 2014; Doria-Rose et al., 2016). This antibody uses a combined mode to engage simultaneously both the C strand and trimer apex hole (Fig. 5 g) (Gorman et al., 2020). A single Fab of all three rhesus antibodies bound asymmetrically to the trimer C3 axis with an extended HCDR3 that penetrated between the N160 glycans of two adjacent protomers (Fig. 5, a, c, and e). Each lineage also recognized N156 glycan from the right-adjacent protomer (perspective from the Fab body toward Env), but to different extents; whereas 6561-DH1020 buried 354 Å^2^ of N156 glycan surface area, V031-a.01 and 40591-a.01 buried only 34 and 89 Å^2^, respectively. The latter two rhesus lineages were like VRC26.25, which buried just 58 Å^2^ of N156 glycan surface area (Table S6).

The HCDR3s from all three lineages recognized the C strand through a combination of sidechain and mainchain interactions (Fig. 5, b, d, and f, top). The V031-a.01 complex revealed the mainchain of Y100j_HCDR3_ to make two parallel strand hydrogen bonds with the mainchain carbonyl and amide of Env residues 167 and 168, respectively, while a string of aromatic residue sidechains (Y100i_HCDR3_, Y100j_HCDR3_, and F100k_HCDR3_) stabilized the extended aliphatic chains of C-strand residues K168, K169, and K171 (Fig. 5 b, top). Further, heavy chain residue E100a_HCDR3_ was positioned such that it could form a salt bridge with K169 from the C strand on the right-adjacent protomer in a manner similar to VRC26.25. Additional C strand interactions with the primary protomer were made by the V031-a.01 light chain: Y30_LCDR1_ hydrogen bonded with K171, and D28_LCDR1_ formed a salt bridge with K168. For 6561-a.01, the mainchains of E100_HCDR3_, Y100a_HCDR3_, and Y100b_HCDR3_ made three antiparallel strand hydrogen bonds with Env residues 169 and 170 (Fig. 5 d, top). Sidechain interactions included the two salt bridges formed between K170 and sulfated Y100b_HCDR3_ and between K171 and E100_HCDR3_, while W100i_HCDR3_ stabilized the extended aliphatic chain of K168. Like V031-a.01, the 6561-a.01 heavy chain residue D100e_HCDR3_ could establish an additional salt bridge with K169 from the C strand on the right-adjacent protomer. Lastly, the 40591-a.01 complex demonstrated that the mainchains of Y100g_HCDR3_, H100h_HCDR3_, and Y100i_HCDR3_ form three parallel-strand hydrogen bonds with Env residues 167 and 169 (Fig. 5 f, top). 40591-a.01 further recognized the C strand through a salt bridge formed between K171 and E100j_HCDR3_; cation–π interactions formed by K168 sandwiched between two His residues (H30_HCDR1_ and H100h_HCDR3_); and stabilization of the K168 aliphatic chain through Y100g_HCDR3_.

Like CAP256–VRC26.25 (also called VRC26.25), the HCDR3 tips of V031-a.01, 6561-a.01, and 40591-a.01 extended beyond the C strand and dipped into the middle of the trimer hole nearly along the C3 symmetry axis (Fig. 5, b, d, and f, bottom). However, unlike VRC26.25, which inserted two sulfated Tyr residues, the rhesus lineages utilized either one (V031-a.01 and 6561-a.01) or none (40591-a.01). Notably, 40591-a.01 lacked a Tyr at the tip of its HCDR3 due to an Y100eF somatic mutation, thereby precluding this posttranslational modification (Fig. 2 b). 6561-a.01 made apex hole interactions most similar to VRC26.25: sulfated residue Y100f_HCDR3_ was positioned to form salt bridges with Env residue 166 from all three protomers (Fig. 5 d, bottom). The V031-a.01 sulfated Y100d_HCDR3_ residue was positioned such that a salt bridge could be formed with a single R166 while mediating cation–π interactions with R166 from a second protomer (Fig. 5 b, bottom). V031-a.01 also inserted two additional anionic residues (D100b_HCDR3_ and D100g_HCDR3_) that formed salt bridges with K169 from two separate protomers; D100g_HCDR3_ could also interact with the third R166 residue. Despite lacking a sulfated tyrosine, 40591-a.01 still engaged apex hole residues from all three protomers (Fig. 5 f, bottom). F100e_HCDR3_ interfaced with V127 and could form cation–π interactions with R166, while D100d_HCDR3_ formed salt bridges with a second R166 residue and K169 from a separate protomer in a manner similar to V031-a.01.

An overlay of these three structures with the Env complex of CAP256–VRC26.25 revealed an approximate alignment of their respective HCDR3s at the simultaneously engaged C strand and apex hole epitopes, but not the Fab bodies themselves (Fig. 5 h and Data S1-Fig. 10 D), providing a structural basis for the ability of both mutations at Env residues 166 and 169 to confer complete neutralization escape from rhesus and human antibodies in this extended class. The center of the VRC26.25 Fab was positioned ∼10-Å further from the Env surface due to its exceptionally long HCDR3 that was 10 to 12 residues longer than each of the rhesus lineage HCDR3s (Fig. 1 b). The relative orientation of the 6561-a.01 heavy and light chains overlapped with VRC26.25, while V031-a.01 and 40591-a.01 were rotated ∼45° and ∼180°, respectively (Data S1-Fig. 10 D). The structure of the V031-a.01 HCDR3 tip was most similar to CAP256–VRC26.25, whereas the inserted aromatic residues of 6561-a.01 and 40591-a.01 were ∼7- and ∼9-Å shallower than VRC26.25, respectively. These data indicate that the defining VRC26 lineage HCDR3 topology does not require exceptional residue length; instead, the resulting distal positioning of the VRC26.25 Fab body likely enables the lack of critical interactions with N160 glycan shared by rhesus lineages (Fig. S2 c and Table S4). Overall, we found chemical and structural mimicry of the human VRC26 lineage to be a reproducible mode of broadly neutralizing V2 apex recognition in rhesus macaques.

### Antibody classes and role of rhesus DH3-15*01

**Figure S5.**
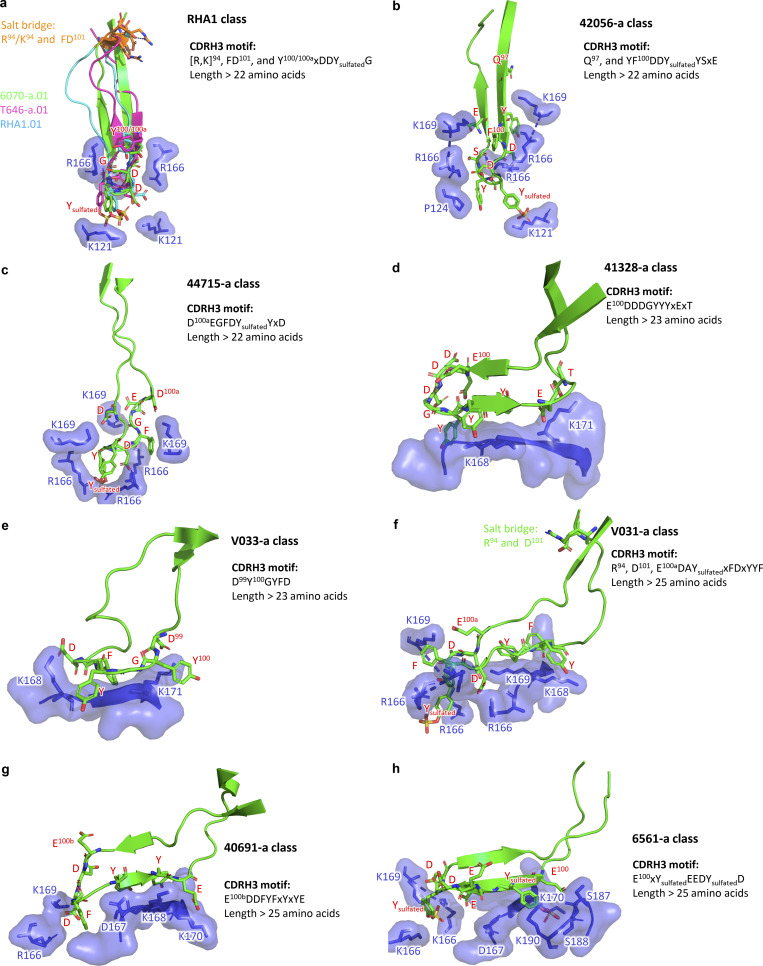
**Structural superimpositions and sequence signatures for rhesus DH3-15–encoded V2 apex antibody classes. (a)** Structural and sequence definition of the RHA1 reproducible antibody class. **(b–h)** Unique structural and sequence definitions of the 42056-a, 44715-a, 41328-a, V033-a, V031-a, 40691-a, and 6561-a antibody classes. In the sequence signatures, “x” represents any amino acid, while the numbers indicate the Kabat positions of the respective residues.

We previously defined antibody classes as antibodies with similar genetic and structural recognition (Kwong and Mascola, 2012; Kwong and Mascola, 2018) and observed some antibodies to form the same class in different individuals (Zhou et al., 2013; Huang et al., 2004). We carried out explicit class analysis of the nine newly determined antibody-Env structures, finding that these segregated into eight separate classes, one of which was reproducible or multi-donor in nature (Fig. S5). The reproducible multi-donor class comprises antibodies from three lineages: lineages 6070-a.01 and T646-a.01 identified in the current study, as well as the previously identified RHA1.01, which achieved ∼50% breadth (Roark et al., 2021). These all utilized a HCDR3 formed with R or K at position 94, F at position 100, D at position 101, and an YxDDYG motif (Fig. S5). Notably, all three members of this multi-donor RHA1-class were elicited by SHIV-CH505 infection (Table S2).

**Figure 6.**
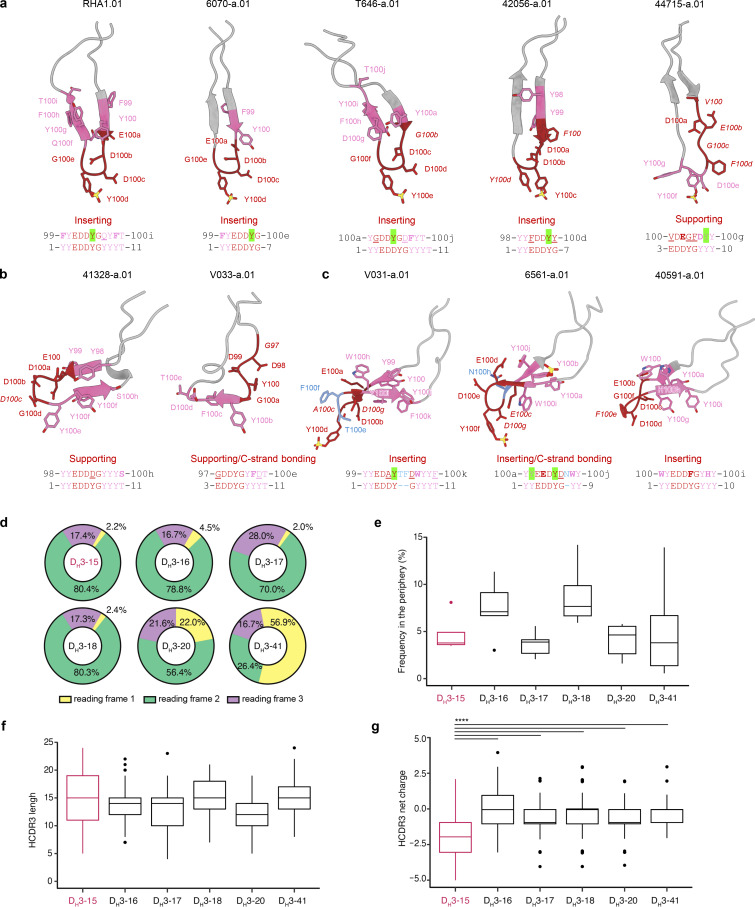
The rhesus DH3-15*01 exhibits structural plasticity and encodes a unique anionic motif. (a) HCDR3 structures from rhesus PGT145-like Fab’s in complex with envelope trimers. DH3-15*01 conserved five-residue motif (EDDYG) positions are colored and labeled in dark red, and the remaining D gene positions are colored and labeled in pink. Conserved motif position labels are italicized when subjected to SHM. D gene position side chains are shown in ball-and-stick representation with nitrogen atoms colored blue, oxygen atoms colored red, and sulfur atoms colored yellow (Kabat numbering). The remaining HCDR3 residues are colored gray with side chains hidden. Below each structure is an alignment of the germline DH3-15*01 coding fragment that was acquired during VDJ recombination (bottom) with the sequence at these positions in the mature antibody (top; Kabat numbering). Somatic mutations that conserve side chain aromaticity or anionic charge are depicted in bold, while discordant somatic mutations are underlined. Sites of tyrosine sulfation are highlighted in green. The functional role of the conserved five-residue motif segment is written above the alignment. **(b)** HCDR3 structures from rhesus PG9-like Fab’s in complex with envelope trimers. Structures are depicted similarly to panel a. **(c)** HCDR3 structures from rhesus VRC26-like Fab’s in complex with SOSIP trimers. Structures are depicted similarly to panel a. Residue insertions in these structures and sequence alignments are colored blue. **(d)** Proportion of reading frame usage among naïve rhesus B cells derived from DH3 genes in the peripheral Indian rhesus macaque repertoire. DH3-15*01 is highlighted in red here and in the remaining panels. B cells derived from rhesus DH3-19*01 are exceedingly rare and therefore excluded from analysis. **(e)** Frequency of DH3 family usage in reading frame two among all naïve B cells in the peripheral rhesus repertoire. **(f)** HCDR3 length distributions of naïve rhesus B cells in the peripheral rhesus repertoire derived from DH3 genes in reading frame two. **(g)** HCDR3 net charge distributions of naïve rhesus B cells in the peripheral repertoire derived from DH3 genes in reading frame two. The net charge of DH3-15*01**–**derived HCDR3s is more anionic (****, P < 0.00001 Student’s *t* test) than all other groups. Charge calculations only consider amino acid residues and not predicted sites of tyrosine sulfation.

To provide a comprehensive view of how DH3-15*01 was able to recapitulate HCDR3-dominated mechanisms of human V2 apex broadly neutralizing antibodies, we evaluated and compared the conformations and interactions of D gene–derived residues of the rhesus lineages that engage prefusion-closed Env (Fig. 6, a–c). The β-turn and one descending or ascending β-strand of all rhesus PGT145-like HCDR3s contained D gene–derived residue positions, with the minimal conserved five-residue motif encompassing the HCDR3 tip in four of five lineages (Fig. 6 a). HCDR3 residues at these positions engaged conserved Env residues 121, 166, and 169 from one or more protomers, most commonly through electrostatic interactions. When these lineages incorporated any of the first two (_1_YY_2_) or last three (_9_YYT_11_) D gene residues, they were left unchanged or somatically mutated to residues that conserved sidechain aromaticity (Fig. 2 b and Fig. 6 a). The four rhesus lineages that inserted their HCDR3s as deeply as human antibody PGT145 preserved the germline-coded _4_DDY_6_ motif, whereas the fifth lineage (44715-a) retained anionic residues at these positions and acquired a similar _8_DY_9_ motif further upstream with a somatic mutation at position eight. Notably, the germline-coded Tyr residue present in either of these motifs was the site of posttranslational sulfation for each lineage (Figs. 3, 5, S4, and S5; and Table S2). The Tyr at position eight was excluded or mutated in all lineages, suggesting this residue to be unfavorable for PGT145-like V2 apex recognition.

The β-strand of the PG9-like HCDR3s that engages the Env C strand is composed of D gene–derived residue positions, while the minimal conserved five-residue motif is positioned in two significantly different conformations (Fig. 6 b). HCDR3 residues at these positions made contacts with Env and glycan residues that are critical for neutralization, but there was not a common pattern of interactions as observed for the lineages with needle-like and combined-mode HCDR3s. Nevertheless, these two lineages each acquired a nine-residue D gene fragment (_3_EDDYGYYT_11_) during VDJ recombination that underwent minimal somatic mutation (Fig. 2 b and Fig. 6 b). As a result, the mature 41328-a.01 and V033-a.01 antibodies each preserved germline-coded _4_DD_5_ and _7_GYY_9_ motifs. Both 41328-a.01 and V033-a.01 lacked tyrosine sulfation (Fig. S4), like human antibody CH03, whose mode of recognition these two rhesus lineages most closely recapitulate.

The inserted HCDR3 tip and one to two β-strands of all combined-mode HCDR3s are composed of D gene–derived residue positions, with the position of the minimal conserved five-residue motif encompassing most or all of the HCDR3 tip for each lineage (Fig. 6 c). HCDR3 residues at these positions comprised a majority of the respective paratopes recognizing both C strand and trimer hole epitopes through interactions with Env residues 166 and 169 on multiple protomers. These three rhesus lineages had each acquired an eight-residue D gene fragment (_1_YYEDDYGY_8_) that underwent minimal somatic mutation, particularly at the N-terminal end (Fig. 2 b and Fig. 6 c). When any of the first two (_1_YY_2_) or last three (_8_YYY_10_) D gene Tyr residues were incorporated, they were left unchanged or somatically mutated to residues that conserved sidechain aromaticity. In addition, all three lineages retained a double anionic _3_ED_4_ or _3_EE_4_ motif. For both V031-a.01 and 6561-a.01, the germline-coded Tyr at position six was retained and bore the posttranslational sulfation. As mentioned above, this Tyr residue was somatically mutated to Phe (Y100eF_HCDR3_) in 40591-a*01, the only VRC26-like antibody that did not exhibit tyrosine sulfation (Fig. 5 f; and Figs. S4 and S5). Interestingly, antibody 40591-a.05 was part of a distinct phylogenetic clade (40591-a.05-08) that retains the germline D gene position six Tyr (Y100e_HCDR3_) (Table S2) and was sulfated as determined by mass spectrometry (Fig. S4).

Thus, residues coded by the DH3-15*01 gene exhibited structural plasticity that enables their incorporation into distinct HCDR3 topologies in otherwise diverse immunogenetic backgrounds to mediate contacts with mode-specific components of the V2 apex epitope.

### Rhesus-specific anionic motif encoded by DH3-15*01 is advantageous for V2 apex recognition

Having elucidated atomic-level interactions between diverse rhesus broadly neutralizing antibodies and the HIV-1 V2 apex, we sought to investigate the immunogenetic basis for the invariant usage of the DH3-15*01 gene by 13 of 13 rhesus antibody lineages (Fig. 2 and Table S2). We first interrogated the rhesus D gene repertoire (Vázquez Bernat et al., 2021) for homologs of the three different D genes from which HCDR3-dominated human V2 apex broadly neutralizing lineages are derived (Fig. S3 d). The rhesus DH3-41*01 gene is a homolog of the human DH3-3*01 gene utilized by the PG9, VRC26, and PCT64 lineages. Alleles of DH3-41*01 bear one to two amino acid substitutions from the 10-residue–long DH3-3*01 gene, with an Asn substitution shared by all alleles resulting in a YYN motif instead of the YYD coded by DH3-3*01; the latter YYD motif is retained in the PG9 and VRC26 lineages, and the terminal Asp residue is retained by PCT64. However, three rhesus non–DH3-3*01 homolog D genes code the same YYD sequence, thus enabling rhesus lineages to acquire this three-residue motif through VDJ recombination. The rhesus DH3-18*01 gene is a homolog of the human DH3-10*01 gene utilized by the CH01 lineage and has substitutions at the C-terminal end. However, since the C-terminal end of DH3-10*01 was excluded from the CH01 lineage VDJ recombination event, rhesus lineages can acquire the same D gene sequence as CH01. Lastly, the rhesus DH4-25*01 gene is a homolog of the human DH4-17*01 gene utilized by the PGT145 lineage. This rhesus D gene bears a single substitution and codes a five-residue DYGNY fragment instead of DYGDY coded by DH4-17*01. PGT145 retains an anionic residue at this position (E100h_HCD3_) that forms a salt bridge with Env residue R166, suggesting this to be an important component of the HCDR3 paratope that rhesus lineages would not acquire through VDJ recombination with this D gene (Fig. 3 e). Notably, all substitutions in rhesus D gene homologs are the result of single-nucleotide polymorphisms and may be readily mutated to match the human germline sequence during affinity maturation. In summary, a lack of homology with human D genes effectively utilized by human V2 apex broadly neutralizing antibodies is likely not responsible for restricting rhesus lineages to DH3-15*01.

**Figure 7.**
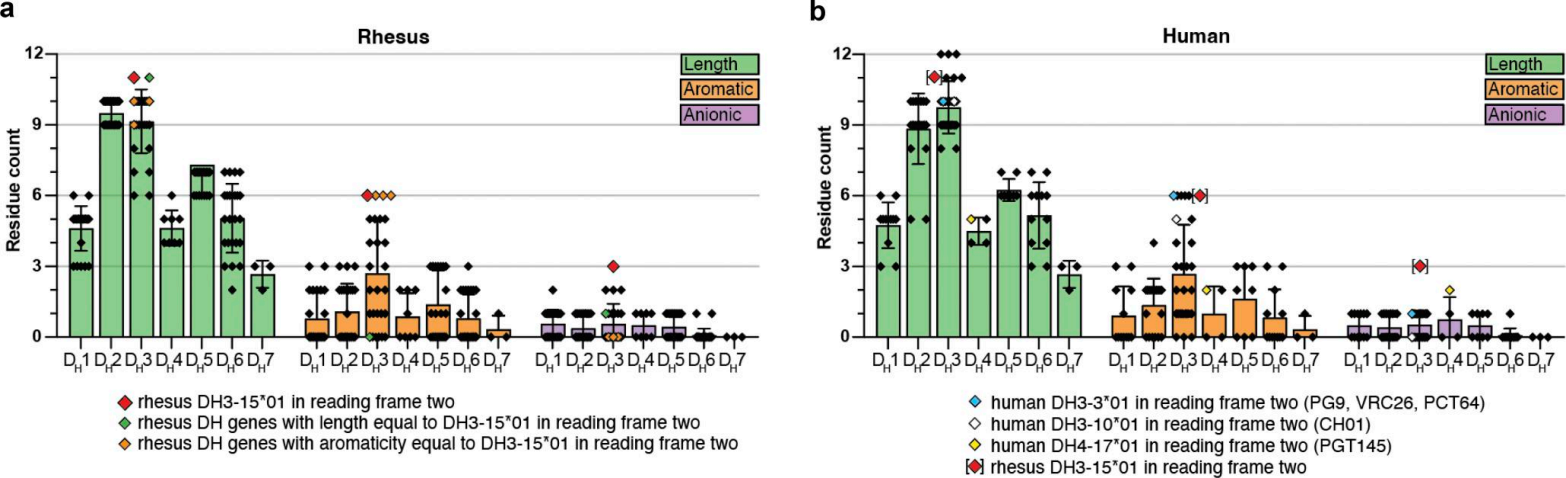
The rhesus DH3-15*01 combines residue features that are advantageous for V2 apex epitope recognition. (a and b) Features of the Indian rhesus macaque and human DH-coding sequence repertoire partitioned by the D gene family. Each dot represents a germline D gene expressed in one of the three forward reading frames with a value corresponding to the total number of residues (length), the number of Tyr, Trp, and Phe residues (aromatic), and the number of Glu and Asp residues (anionic). Boxes extend to the respective D gene family mean value, and whiskers show the standard deviation. Sequences were excluded if stop codons were found in the middle of the coding sequence in any particular reading frame. In panel a, the rhesus DH3-15*01 gene expressed in reading frame two is marked with a red symbol, and rhesus D genes with length and aromatic features equal to DH3-15*01 in reading frame two are marked with green and orange symbols, respectively. In panel b, D genes utilized by HCDR3-dominated human V2 apex lineages are marked according to the legend, and the rhesus DH3-15*01 gene expressed in reading frame two is included as a reference to facilitate comparison.

We next interrogated the rhesus D gene repertoire for sequence characteristics shared by V2 apex–targeted rhesus and human lineage HCDR3s: length, aromatic residues, and anionic residues (Fig. 7 a). Similar to the human D gene repertoire, the rhesus DH2 and DH3 families are comprised of genes with some of the longest nucleotide lengths, a majority of which could contribute up to 10 residues in at least one reading frame. The longest D gene fragments in these families were of 11 residues, which were expressed by IGHD3-15*01 in reading frame two and IGHD3-20*01 in reading frame three. The DH3 family was also comprised of the genes coding for the greatest number of aromatic residues in the D gene repertoire, which ranged from four to six residues in reading frame two. Finally, we found that DH3-15*01 alone expresses three anionic residues in reading frame two, which is the greatest number in the D gene repertoire. The next highest number was two anionic residues expressed in reading frame two by DH1-7*01, DH3-17*01, DH3-19*02_S4720, and DH3-20*01. To assess biochemical properties favorable for V2 apex recognition expressed by rhesus DH3-15*01 versus human D genes, we next analyzed the rhesus DH3-15*01 gene relative to translated human D genes (Fig. 7 b). We observed the rhesus DH3-15*01 in reading frame two to be at the extreme for aromaticity and negative charge compared with human D genes. With length, however, at 11 residues, it was shorter than three human D genes of up to 12 residues. Of note, the one human D gene sequence with two anionic residues is DH4-17*01 in reading frame two, which is the same gene and reading frame used by the PGT145 lineage; this gene, however, is only five residues long (DYGDY). Overall, we found that the rhesus DH3 family contains multiple genes that combine two or more sequence features favorable for V2 apex recognition despite the exclusive use of DH3-15*01 by rhesus lineages.

To investigate any functional VDJ recombination biases that could explain this phenomenon, we analyzed the features of DH3-derived HCDR3s in the peripheral rhesus B cell repertoire. DH3 family genes must be expressed in reading frame two to code for their characteristic anionic and aromatic residues and be devoid of stop codons. Approximately 80% of naïve B cells derived from DH3-15*01 incorporated this gene in reading frame two, which was a similar frequency to those derived from DH3-16*01, DH3-17*01, and DH3-18*01 (Fig. 6 d). Naive B cells derived from DH3-15*01 in reading frame two were not overrepresented in the periphery, nor did they have substantially longer HCDR3s than B cells expressing other DH3 family genes (Fig. 6, e and f). However, the net HCDR3 charge distribution of DH3-15*01–derived B cells was significantly more anionic (P < 0.00001) (Fig. 6 g).

While single-component analysis showed the unique three-residue anionic motif expressed by DH3-15*01 in reading frame two to be the largest statistical outsider for the predilection of rhesus V2 apex–targeted lineages to utilize this D gene, it may be the combination of length, aromaticity, and negative charge that coalesce to make this D gene so highly overrepresented. DH3-15*01 in its second reading frame was an outlier compared with nearly all other D genes in length, aromaticity, and net negative charge (Fig. 7 a). We note in this context that these characteristics are all favorable for tyrosine sulfation (Huttner, 1982; Monigatti et al., 2002; Moore, 2003), which is utilized in many of the inserting HCDR3 motifs observed in rhesus (Figs. 3, 5, 6, and S4) and human V2 apex–targeted broadly neutralizing antibodies (Pejchal et al., 2010; Mclellan et al., 2011; Lee et al., 2017; Rantalainen et al., 2018; Gorman et al., 2020; Cai et al., 2022). In summary, the unique and favorable biochemical properties of DH3-15*01 in reading frame two likely led to it being highly selected in rhesus macaques irrespective of the mode of antibody recognition.

### Comparison of rhesus and human neutralizing antibody recognition of the HIV-1 V2 apex site of vulnerability

**Figure 8.**
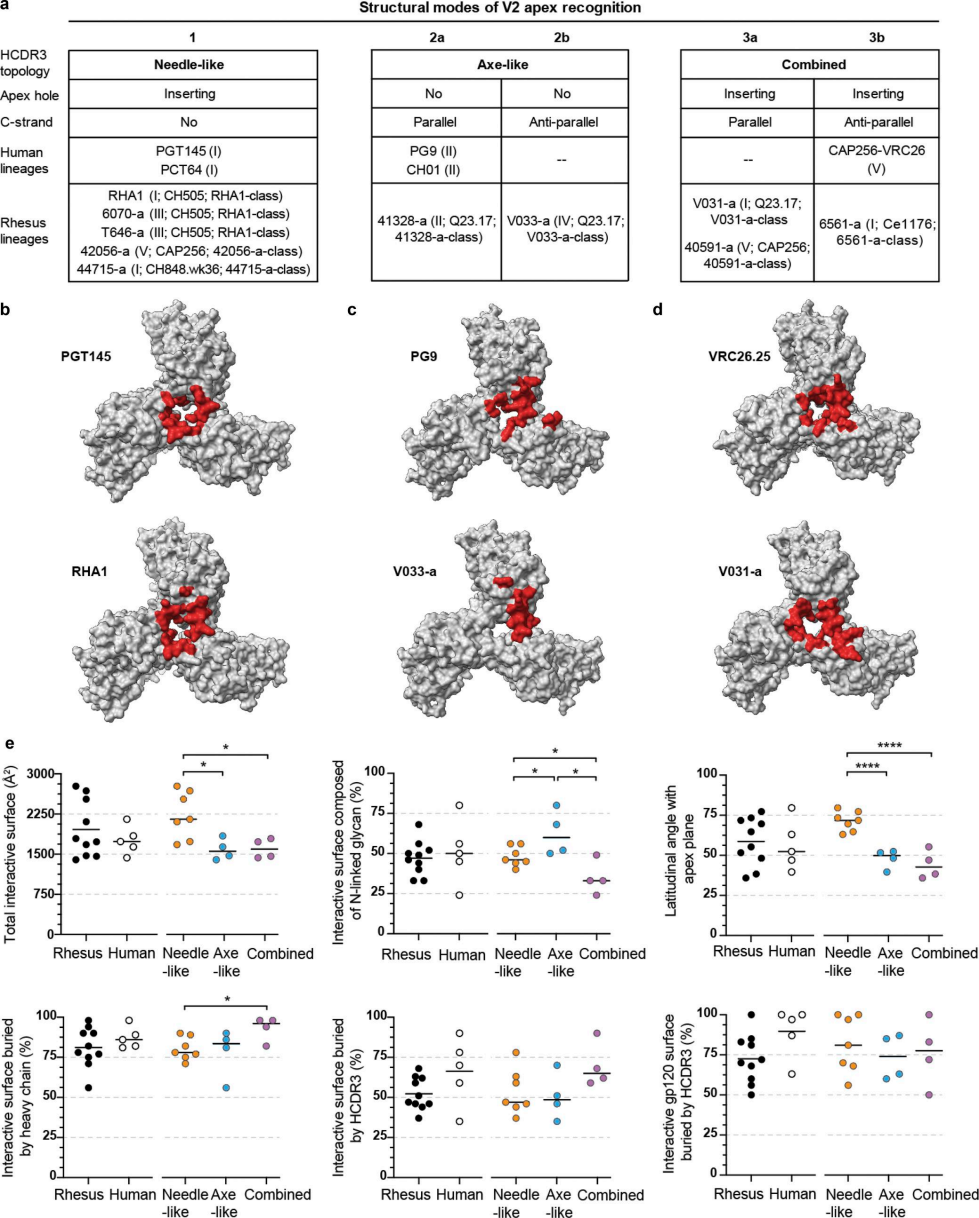
**Cross-species antibody recognition of the HIV-1 V2 apex site of vulnerability and their induction by SHIV. (a)** Summary of rhesus and human structural modes of HCDR3-dominated V2 apex recognition. Roman numerals denote the neutralization mapping group (groups I**–**V) for each lineage as detailed in extended data Fig. 2 D; infecting SHIV strain and antibody class are also delineated as detailed in extended data Table S2 and extended data Fig. 6, respectively. **(b–d)** The 5-Å Env footprints of the most broadly neutralizing rhesus (top) and human (bottom) lineages with needle-like (b), axe-like (c), and combined (d) modes of recognition are mapped in red onto each respective trimer complex. The remaining gp120 surface is shown in gray, and all other components of the structure are omitted for clarity. Top view of trimer. **(e)** Epitope and paratope characteristics of V2 apex lineages compared across species and extended class. Each plot is a different structural feature that groups lineages by host species on the left and HCDR3 topology on the right. P values are only listed for statistically significant differences (*, P < 0.05; ***, P < 0.0005; unpaired *t* test) between two groups on the same side of the plot.

To further define modes of V2 apex recognition shared by rhesus and human broadly neutralizing antibodies, we analyzed features of both their structural architecture and their interactive surfaces. For evaluating structural interactions, we used apex hole and C-strand recognition to categorize human and rhesus antibodies into recognition modes, which showed little concordance with neutralization mapping groups (Fig. 8 a and Fig. S2 c). However, all antibodies utilizing structural modes that insert into the apex hole were sensitive to mutations at Env residue 166, whereas antibodies with non-inserting structural modes were unaffected (Fig. S2 c and Table S4). There was significant overlap of the Env surfaces recognized by the most broadly neutralizing lineages across all classes with modest variation between species (Fig. 8, b–d). The PG9-like lineages had the smallest footprint on Env, while PGT145-like and VRC26-like lineages appeared indistinguishable except for the deeper PGT145-epitope surface obstructed within the trimer. The average total interactive surface area (protein and *N*-linked glycan epitopes) of PGT145-like lineages was greater than both PG9-like and VRC26-like, which were nearly identical to each other (Fig. 8 e and Table S6). *N*-linked glycans comprised the greatest fraction of the total interface surface for PG9-like antibodies (50% or greater for all lineages) and the least for VRC26-like antibodies. Rhesus and human antibodies utilized mode-specific angles of approach to recognize these surfaces: PGT145-like lineages consistently recognized the apex surface with a much steeper approach than PG9-like and VRC26-like antibodies. Similar angles of approach for the latter two recognition modes suggest a restricted spatial freedom to penetrate the apical glycan shield and form mainchain β-strand interactions with the Env C strand. The heavy chain comprised a majority of the paratope for all lineages, a substantial fraction of which was specifically mediated by HCDR3. Human lineages tended to have more HCDR3-dominated recognition, but this difference was not statistically significant. Overall, structural features of V2 apex recognition appeared to be mode-specific and conserved across species.

## Discussion

In this study, we isolated 11 V2 apex–directed antibody lineages with cross-clade neutralization breadth from 10 SHIV-infected Indian-origin rhesus macaques. Relative to other categories of broadly neutralizing antibody, we observed generally lower SHM for these macaque antibodies relative to their breadth, with antibody V033-a.01 having 37% breadth (208-strain panel) and <5% (nucleotide) SHM. Correlations between isolated antibodies and plasma neutralization indicated a single V2 apex–neutralizing lineage to account for the cross-clade breadth in most macaques. Cryo-EM structures of Fab–Env complexes for nine rhesus lineages revealed modes of recognition that included, and expanded on, three canonical modes of human-V2 apex antibody recognition. Despite this substantially increased topological diversity of HCDR3, all SHIV-elicited V2 apex lineages utilized the same DH3-15*01 gene in reading frame two.

In prior cases of highly selected genetic elements, such as VH1-69 in influenza stem antibodies (Sui et al., 2009; Dreyfus et al., 2012), VH1-2 together with a 5-amino acid LCDR3 in HIV-1 VRC01-class CD4-binding antibodies (Wu et al., 2011; Zhou et al., 2013), or DH3-22 directed to the cryptic site of the sarbecovirus receptor-binding domain (Liu et al., 2022; Yuan and Wilson, 2024), the highly selected genetic elements each utilize the same structural mode of recognition. This contrasts with the results of the current study in which the highly selected rhesus DH3-15*01 gene, a clear signature for V2 apex–directed broadly neutralizing antibodies, encoded amino acids that played diverse structural roles: sometimes as an inserting loop, sometimes as a contacting β-strand, and sometimes as part of the supporting non-contacting HCDR3 loop. In these diverse structural roles, multiple modes of V2 apex recognition were observed in which each recognition mode forms an extended class, which we previously defined as antibodies that did not necessarily share genetic commonalities but nonetheless displayed a characteristic mode of antigen interaction (Gorman et al., 2016). Remarkably, DH3-15*01 plays diverse structural roles not only between modes but also within the same extended class.

**Figure 9.**
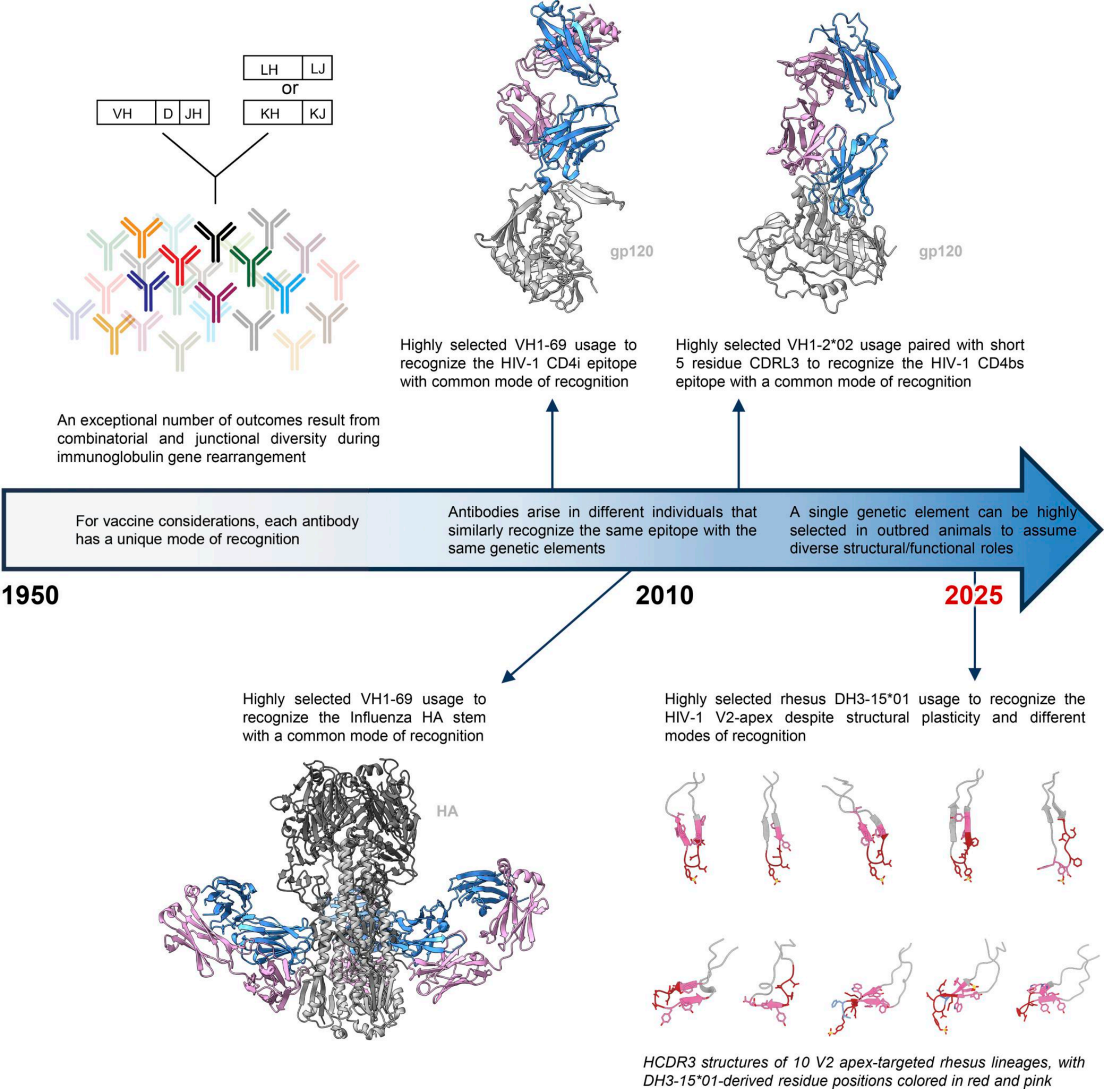
**Highly selected immunogenetic elements can adopt diverse structural and functional roles in epitope recognition.** A timeline of the vaccine field’s evolving understanding of antibody development and specificity. It was initially thought that the high diversity of possible antibodies generated from V(D)J recombination and heavy-light chain pairing meant that, for vaccine purposes, each generated antibody would be unique, and antibodies in different individuals would be different. However, from 2004 to 2013, several studies established that virtually identical antibodies could arise in different individuals due to genetic elements being highly selected to recognize the same HIV-1 gp120 or influenza HA epitopes through similar, often identical, structural modes of recognition. Thus, the same antibodies could arise in different individuals with genetic elements selected to have the same mode of recognition. Now, we have discovered that it is possible for a highly selected genetic element to adopt different modes of recognition – with the selected genetic element exhibiting both structural diversity and distinct recognition chemistries.

We propose that it is the biochemical properties of the D gene, specifically its length, aromaticity, and charge, which are the most critical determinants of V2 apex recognition. This, coupled with the observed structural diversity of the rhesus DH3-15*01 gene, provide a mechanistic explanation for its extremely frequent, if not invariant, “signature” usage in rhesus V2 apex broadly neutralizing lineages. The ability of highly selected genetic elements to assume divergent functional roles—an ability to our knowledge not previously reported—expands our understanding of the determinants of antigen recognition, with consequences related to antibody diversity and multi-donor reproducibility (Fig. 9).

The striking frequency of V2 apex broadly neutralizing antibodies using the DH3-15*01 gene, with its favorable germline-encoded EDDYG motif, in SHIV-infected rhesus macaques has important implications for the preclinical testing of V2 apex–targeted vaccines: specifically, it suggests that the elicitation of V2 apex broadly neutralizing antibodies may be easier to achieve in nonhuman primates than in humans, which do not have a homologous EDDYG-encoding D gene. That said, the frequency of EDDYG-containing HCDR3s in humans may be more common than initially presumed since this motif can be generated by non-templated N-nucleotide addition at the VH V–D junction (Habib et al., 2024, *Preprint*). We note that the repeated elicitation of V2 apex broadly neutralizing antibodies in rhesus macaques by SHIV infection provides a model system to elucidate essential elements of V2 apex broadly neutralizing antibody induction by candidate vaccines.

It will be fascinating to see how much the frequency of elicitation of V2 apex broadly neutralizing antibodies can be improved by novel germline-targeted immunogens currently under development (Melzi et al., 2022; Willis et al., 2022; Habib et al., 2024, *Preprint*) and if a combination of vaccine priming followed by SHIV infection can enhance the induction of high titer serological neutralization, as recently demonstrated for the fusion peptide site of vulnerability (Wang et al., 2024). In this context, it will be important to decipher key elements in Env–antibody coevolution in SHIV-infected macaques that are responsible for guiding affinity maturation to engender neutralization breadth and potency (Habib et al., 2025, *Preprint*) and how these steps can be replicated by rational vaccine design (Haynes et al., 2012; Haynes et al., 2023; Saunders et al., 2019; Caniels et al., 2023; Wiehe et al., 2024). Such analyses should be able to decipher why select SHIV strains may preferentially induce specific modes of V2 apex recognition, since all three macaques that were infected with SHIV-CH505 developed “needle” responses (structural mode 1 of V2 apex recognition), whereas all three macaques infected with SHIV-Q23.17 showed C-strand recognition, which was parallel, antiparallel, or combined in nature (structural modes 2a, 2b, or 3 of V2 apex recognition). Critical insight gained from the fuller repertoire of V2 apex–directed broadly neutralizing antibodies provided here may ultimately enable such antibodies to be elicited by vaccination alone.

## Materials and methods

### Nonhuman primates

All Indian rhesus macaques used in this study were housed at Bioqual, Inc., Rockville, MD, according to guidelines of the Association for Assessment and Accreditation of Laboratory Animal Care. Experiments were approved by the University of Pennsylvania (IACUC protocol 806719) and Bioqual (IACUC protocol 21-139) Institutional Animal Care and Use Committees. Macaques were sedated for blood draws, anti-CD8 mAb infusions, and SHIV inoculations and cared for according to AAALAC guidelines and best practice standards. The 10 rhesus macaques described in this study were as follows: 5695 (M, 6 yo), 6561 (M, 7 yo), 6070 (M, 4 yo), 40591 (M, 6 yo), 42056 (M, 5 yo), T646 (F, 5 yo), V031 (F, 4 yo), V033 (F, 3 yo), 41328 (M, 6 yo), and 44715 (F, 4 yo). Rhesus macaques 42056, V033, V031, 44715, and 6070 were transiently depleted of CD8 T cells prior to SHIV inoculation with a 25–50 mg/kg subcutaneous or intravenous infusion of anti-CD8α (MT807R1) or anti-CD8β (CD8beta255R1) mAb (NIH Nonhuman Primate Reagent Resource, https://www.nhpreagents.org). Rhesus macaques were inoculated intravenously with 293T transfection supernatants containing 50 or 500 ng p27 Ag of molecularly cloned SHIV challenge stock, except for RM 5695, which was inoculated with acute-phase SHIV-infected rhesus plasma as previously described (Roark et al., 2021). SHIVs used to infect each animal are listed in Fig. S1 and Table S2. Design features of SHIVs and methods for preparing 293T-derived virus challenge stocks were previously described (Li et al., 2021). Infection of rhesus macaques 5695, 6070, 40591, and 42056 was previously reported by Roark et al. (2021). Infection of rhesus macaque T646 was previously reported by Bauer et al. (2023). Rhesus macaques 5695, V033, and V031 were repurposed from previous HIV-1 Env DNA or SOSIP immunization studies (animal IDs 5695, 177715, and 178951, respectively) (Williams et al., 2017; Han et al., 2020). All repurposed animals lacked detectable tier-2 neutralizing responses in plasma prior to infection. Rhesus macaque 44715 was infected with SHIV CH848.wk36con, a consensus sequence of plasma viruses at 36 wk after infection from a SHIV CH848-infected rhesus macaque. SHIV plasmids used in these experiments have been deposited in the American Type Culture Collection under contract with the National Institute of Allergy and Infectious Diseases (NIH).

### Blood processing

Blood samples were collected in sterile vacutainers containing acid citrate dextrose formula A anticoagulant. Acid citrate dextrose formula A blood (40 ml) was centrifuged (1,000 *g* for 10 min at 20°C) in sterile 50-ml conical tubes, and the plasma was collected without disturbing the buffy coat white blood cell layer and red cell pellet. Plasma was centrifuged again (1,500 *g* for 15 min at 20°C) to remove platelets, aliquoted into 1-ml cryovials, and stored at −80°C. The cell pellet was resuspended in an equal volume of HBSS (without Ca/Mg) containing 2 mM EDTA and divided into four 50-ml conical tubes. Additional HBSS-EDTA buffer was added to each tube to bring the total volume to 30 ml. The cell suspension was underlayered with 14 ml of 96% Ficoll-Paque and centrifuged (725 *g* for 20 min at 20°C) with slow acceleration and braking. Mononuclear cells at the Ficoll interface were collected and transferred to a new 50-ml centrifuge tube containing HBSS-EDTA and centrifuged (200 *g* for 15 min at 20°C). The supernatant was removed, and the cell pellet was resuspended in 40 ml HBSS (with Ca/Mg) + 1% FBS. The suspension was centrifuged again (200 *g* for 15 min at 20°C), and the supernatant was discarded. Centrifugation at 200 *g* pellets white blood cells but allows most platelets to remain in suspension. The mononuclear cell pellet was tap resuspended in the residual media, and then HBSS (with Ca/Mg) + 1% FBS was added to a volume of 10 ml. Cells were counted, and viability was assessed by trypan blue staining. Cells were centrifuged again (300 *g* for 10 min at 20°C), the supernatant discarded, and the cells resuspended at a concentration of 5–10 × 10^6^ cells/ml in CryoStor cryopreservation media (cat. no. C2999; Sigma-Aldrich) and aliquoted into 1-ml cryovials (CryoClear cryovials; cat. no. 3010; Globe Scientific Inc.). Cells were stored in a Corning CoolCell LX cell-freezing container at −80°C overnight and then transferred to vapor-phase liquid nitrogen for long-term storage.

### Site-directed mutagenesis

Site-directed mutants in this study were generated using a Q5 site-directed mutagenesis kit according to the manufacturer’s instructions (NEB). Briefly, mutagenesis primers were designed using the NEBaseChanger (https://nebasechanger.neb.com/) to contain a mismatch at the residue of interest. The template virus was then PCR amplified with the mutagenesis primers, and the resulting PCR was product treated with KLD enzyme mix to remove template DNA and ligate blunt PCR ends. Plasmids were then transformed into *Escherichia coli* DH5α cells (for Env clones) or MAX Efficiency Stbl2 cells (for full-length SHIV clones) and grown overnight at 30°C. Plasmid preparations were sequence verified to confirm the presence of the desired mutation.

### Virus stocks

SHIV and pseudovirus stocks were prepared by transfection of HEK 293T/17 cells (CRL-11268; the American Type Culture Collection). 4–5 × 10^6^ cells were plated in 100-mm dishes in DMEM containing 10% FBS and 1% penicillin/streptomycin and incubated overnight at 37°C. 6 µg of plasmid DNA was then transfected using FuGENE 6 transfection reagent (Promega) according to the manufacturer’s instructions. Transfected cells were then incubated for 48 h at 37°C. Supernatants were centrifuged (2,000 *g* for 8 min at 4°C) to remove cell debris, aliquoted, and stored at −80°C. Viruses were titered on TZM-bl cells. Seven fivefold serial dilutions of virus stocks were made in DMEM with 6% FBS and 40 µg/ml DEAE-dextran, added in quadruplicate to adherent TZM-bl cells, and incubated at 37°C for 48 h. Following incubation, cells were fixed for 10 min at room temperature in PBS containing 0.8% glutaraldehyde and 2.2% formaldehyde. Cells were then washed three times with PBS and stained with PBS containing 4 µM magnesium chloride, 4 µM potassium ferricyanide, 4 µM potassium ferrocyanide, and 400 µg/ml X-Gal for 3 h at 37°C. Stained cells were then washed three times with PBS and imaged on a CTL Immunospot analyzer.

### Neutralization assays

Neutralization assays were performed on TZM-bl indicator cells as previously described (Roark et al., 2021). Briefly, TZM-bl cells were plated in 96-well plates in DMEM containing 10% FBS and 1% penicillin/streptomycin and grown overnight at 37°C. Serial plasma or mAb dilutions were incubated the with virus at 37°C for 1 h. Plasma was diluted in cell culture media containing 5% normal human or rhesus heat-inactivated serum so as to hold the concentration of test plasma/serum constant across all wells. Following incubation, the virus/antibody mixture was added to TZM-bl cells, and the cells were incubated for 48 h at 37°C. Cells were then lysed with PBS containing 0.5% Triton X-100 at room temperature for 1 h, and luciferase levels were quantified using the Promega Luciferase Assay (Promega) on a BioTek Synergy Neo2 microplate reader.

### Rhesus mAb isolation

Broadly neutralizing antibody lineages were isolated from memory B cells baited with antigen-specific probes through single-cell sorting into PCR plates. PBMCs were thawed, washed in 10 ml RPMI + 10% FBS + 2 μl RNase-free DNase I (NEB), and stained with LIVE/DEAD Aqua for 15 min at room temperature. PBMCs were then washed twice with PBS and stained with a cocktail of CD3-PerCP-Cy55, CD4-BV785, CD8-BV711, CD14-PE-Cy7, CD20-BV605, IgD-FITC, IgG-AF680, and IgM-BV650 for 15 min at room temperature. PBMCs were again washed twice with PBS and then stained with fluorophore-conjugated SOSIP probes (detailed in Table S2) for 15 min at room temperature. Memory B cells (CD3^−^, CD4^−^, CD8^−^, CD14^−^, CD20^+^, IgG^+^, IgD^−^, and IgM^−^) positive for wild-type heterologous SOSIP probes and negative for V2 apex epitope mutant probes were sorted at 1 cell per well into 96-well plates on a BD FACSAria II machine using BD FACSDiva software (BD Biosciences). RNA extraction, cDNA synthesis, and VDJ gene amplification were performed as previously described (Mason et al., 2016). Wells with successful DNA PCR amplification were Sanger sequenced (Azenta Biosciences) and initially analyzed with IgBLAST (Ye et al., 2013) or IMGT V-QUEST (Brochet et al., 2008) to identify expanded lineages and inspect HCDR3 sequences.

### Bone marrow–negative selection

For isolation of antibody 5695-b.01, RM5695 necropsy (week 65) bone marrow aspirate mononuclear cells were thawed, washed in 10 ml RPMI + 10% FBS + 2 μl RNase-free DNase I (NEB), and placed in single-cell suspension in 1 ml RPMI + 10% FBS. First, 25 μl of Human TrueStain TcX (cat # 422301; BioLegend) was added to the cell suspension and incubated for 10 min at room temperature. To enrich for plasma cells, the total mononuclear cells were then incubated with antihuman CD3-PerCP-Cy55, CD4-BV785, CD8-BV711, CD14-PE-Cy7, CD20-BV605, CD16, and CD36 for 20 min at 4°C under gentle agitation. Following two wash steps in RPMI + 10% FBS, cells were resuspended in 100 μl of autoMACS Running Buffer (Miltenyi Biotec), and 25 μl of anti-mouse IgG microbeads (Miltenyi Biotec) were added, followed by a 15 min incubation at 4°C. After two wash steps with autoMACS Running Buffer, cells were resuspended in 500 μl of autoMACS Running Buffer and loaded on an LS Column (Miltenyi Biotec) for magnetic separation using a QuadroMACS Separator (Miltenyi Biotec), following the manufacturer’s protocol. Flow-through cells were pelleted and resuspended in 250 μl of RPMI + 10% FBS. Cell viability was analyzed using AO/PI staining using a Cellometer Auto 2000 Cell Viability Counter (Nexcelom Bioscience) and determined to be 83.7%.

### Single-cell B cell receptor sequencing

Negatively selected bone marrow cell suspension was loaded on a Chromium X instrument (10X Genomics) to generate single-cell bead emulsion at a loading concentration for a targeted recovery of 10,000 cells per reaction. Single-cell RNA-seq libraries were then prepared using the Chromium Next GEM Single Cell 5′ Kit v2 bead and library construction kit (10X Genomics). B cell receptor (BCR) libraries were constructed using the Chromium Single Cell Human BCR Amplification Kit (10X Genomics) and rhesus macaque–specific primers targeting the constant regions of heavy chain IgM, IgG, and IgA gene isotypes and light chain IgK and IgL genes, as described previously (Han et al., 2020). BCR libraries were indexed using a Dual Index Kit TT Set A kit (10X Genomics) and sequenced on an Illumina NextSeq 2000 instrument at a minimum read depth of 5000 reads/cell. Illumina BCLconvert 3.10.12 was used for demultiplexing, and fastq files were analyzed with Cell Ranger 7.2.0’s VDJ pipeline (10X Genomics) using a custom rhesus macaque VDJ germline reference. The 5695-b.01 mAb was identified based on manual analysis of HCDR3 length, sequence homology, and heavy/light chain germline gene usage.

### mAb cloning and synthesis

Antibodies with features characteristic of V2 apex broadly neutralizing antibodies (expanded lineages with long CDRH3s enriched for negative and aromatic residues) were selected for further analysis. Antibody heavy chain VDJ and light chain VJ gene cassettes were synthesized (Genscript) and cloned into rhesus IgG1 (RhCMV-H), IgK (RhCMV-K), or IgL (RhCMV-L) expression vectors upstream of their constant regions using AgeI/NheI, AgeI/BsiWI, and AgeI/ScaI restriction sites, respectively (Mason et al., 2016). Plasmids encoding antibody heavy and light chain genes were used to transfect suspension Expi293F cells in a 1:2 heavy-to-light chain ratio using ExpiFectamine 293 transfection reagents (Gibco) according to the manufacturer’s instructions. Antibodies were purified using an rProtein A/Protein G Gravitrap kit (Cytiva), buffer exchanged into PBS, and stored at 4°C.

### B cell next-generation sequencing

PBMCs were stained with LIVE/DEAD Aqua, CD3-PerCP-Cy55, CD4-BV785, CD8-BV711, CD14-PE-Cy7, CD20-BV605, IgD-FITC, IgG-AF680, and IgM-BV650. Naïve B cells (CD3^−^, CD4^−^, CD8^−^, CD14^−^, CD20^+^, IgG^−^, IgD^+^, and IgM^+^) and memory B cells (CD3^−^, CD4^−^, CD8^−^, CD14^−^, CD20^+^, IgG^+^, IgD^−^, and IgM^−^) were bulk sorted into RPMI with 10% FBS and 1% penicillin-streptomycin using a BD FACSAria II sorter. Total RNA was extracted using RNAzol RT as per the manufacturer’s guidelines (Molecular Research Center, Inc.). Reverse transcription of mRNA transcripts, IgM and IgL variable region library preparation, and next-generation sequencing were performed as previously described (Krebs et al., 2019; Roark et al., 2021). Briefly, cDNA was synthesized using a 5′RACE approach, with SMARTer cDNA template switching and Superscript II RT, following which cDNA was purified using AMPure XP beads (Beckman Coulter). cDNA was then PCR amplified using KAPA HotStart ReadyMix (Roche) with IgG, IgM, IgK, or IgL constant region–specific primers to amplify B cell heavy chain VDJ regions and light chain VJ regions. Finally, the libraries were PCR amplified with primers that added on barcodes and Illumina P5 and P7 sequencing adapters. Both heavy and lambda immunoglobulin libraries were sequenced on an Illumina MiSeq sequencer with 2 × 300-bp runs using the MiSeq Reagent V3 kit (600 cycle). Demultiplexed reads were adapter trimmed using Cutadapt v3.5, and paired forward/reverse reads were merged using PEAR v0.9.6 with a quality cutoff of 15 and a minimum length of 50.

### VDJ repertoire analysis

Filtered, quality-controlled IgM and IgL sequences were analyzed using IgDiscover v0.15.1 (Corcoran et al., 2016) to curate a personalized immunoglobulin repertoire library for each rhesus macaque (Table S3). Merged reads were used as input, with the KIMDB 1.1 database (Vázquez Bernat et al., 2021) (http://kimdb.gkhlab.se/) used as the heavy chain reference and the Ramesh et al. (2017) allele database used as the light chain reference. The IgDiscover J output for both heavy and light chains was additionally filtered with the “discoverjd” feature (using a J coverage setting of 100 and allele ratio of 0.33) to exclude low-confidence novel alleles. This allowed the curation of a repertoire of both known and novel V and J gene alleles for each rhesus macaque, confirmed the presence of known D gene alleles, and allowed the accurate assignment of V, D, and J alleles for each broadly neutralizing antibody lineage. Data from IgG sequencing datasets were analyzed using SONAR v4.2 (Schramm et al., 2016). The manually guided identity/divergence feature was used to identify ancestral clonal sequences. As IgDiscover does not identify novel D gene alleles, per-animal D gene repertoires were further investigated with MINING-D (Bhardwaj et al., 2020). Deduplicated HCDR3 sequences of SONAR-clustered reads were used as input, allowing the detection of more complete D segments. These were then compared with published references (Corcoran et al., 2016; Ramesh et al., 2017; Vázquez Bernat et al., 2021) to further verify the presence of known D gene alleles in each animal.

### Neutralization heatmap and clustering analyses

IC_50_ neutralization titers for prototypic human broadly neutralizing antibodies for the 119-virus panel were obtained from CATNAP on the Los Alamos HIV Databases (https://www.hiv.lanl.gov). This dataset, together with the rhesus V2 apex antibody dataset generated here, was subjected to analysis with the Heatmap webtool on the Los Alamos HIV Database (options used were: Log10 transformation, Euclidean distance, complete Ward hierarchical clustering algorithm, and 100 bootstrap replicates). For correlation clustering analyses, the “corrplot” package (version 0.95) was used on the same dataset as above. Pearson correlation of log_10_-transformed IC_50_ titers between each pair of broadly neutralizing antibodies, by using a vector of 119 pseudovirus IC_50_ titers to characterize each antibody. Automated hierarchical clustering was performed with the complete Ward clustering algorithm used to ensure comparisons with the above.

### Prefusion-stabilized envelope trimer production

All recombinant HIV-1 and SIVcpz envelope SOSIP trimers used for antigen-specific single-cell sorting and cryo-EM structure analysis were designed, expressed, and purified using previously described methods (Sok et al., 2014; Kwon et al., 2015; Doria-Rose et al., 2016; Steichen et al., 2016; Saunders et al., 2017; Andrabi et al., 2019; Rawi et al., 2020; Wrapp et al., 2023). Plasmids encoding the SOSIP trimer construct and human furin were mixed at a 4:1 ratio and complexed with 293fectin (Thermo Fisher Scientific) to transfect 293F cells with ∼0.6 mg DNA/1 L of suspension cell culture. 6 days after transfection, cultures were clarified by centrifugation, and native, prefusion-closed trimers were purified from culture supernatant using affinity chromatography with immobilized PGT145. Gel filtration on a Superdex 200 16/600 column (GE Healthcare) was used to further purify and buffer exchange each trimer into PBS. Trimers to be used as probes for single-cell sorting were appended with a C-terminal Avi-tag (GGLNDIFEAQKIEWHED) to enable biotinylation using BirA biotin-protein ligase (Avidity) once purified. Streptavidin-linked fluorophores (SA-PE, SA-BV421, or SA-APC) were added to biotinylated trimers for 75 min at 4°C to prepare them as multimerized memory B cell probes.

### Antibody Fab production

Fab’s were expressed and purified with one of two approaches. One approach utilized was to insert the HRV3C protease cleavage site (LEVLFQGP) into the hinge region of the plasmid encoding the full-length rhesus IgG1 heavy chain. Antibodies bearing the HRV3C cleavage site were expressed and purified as described above by swapping in the modified heavy chain plasmid to be paired with the plasmid encoding the natural light chain. 1 mg of antibody was digested with HRV3C protease (Thermo Fisher Scientific) for ∼16 h at 4C, from which liberated Fab fragments were purified using negative selection on a Protein A column. The second approach utilized was to insert an 8× histidine tag and an early stop codon into the hinge region of the plasmid encoding the full-length rhesus IgG1 heavy chain. When co-transfected with the plasmid encoding the natural light chain mixed at a 1:1 ratio, Fab was purified from clarified culture supernatant 6 days after transfection using Ni-NTA affinity chromatography with IMAC Sepharose 6 Fast Flow resin (GE Healthcare). Gel filtration on a Superdex 75 10/300 column (GE Healthcare) was used to further purify and buffer exchange each Fab into PBS.

### Cryo-EM sample preparation

Fab’s and HIV-1 envelope SOSIP trimer complexes were prepared for cryo-EM data collection as previously described (Lee et al., 2017; Gorman et al., 2020; Roark et al., 2021). Briefly, envelope trimers were concentrated to ∼4 mg/ml and mixed with Fab at a 1:3 M ratio for a final trimer concentration of ∼2–3 mg/ml. The mixture was incubated on ice for 20 min to allow complexes to form before the addition of the detergent n-Dodecyl β–D-maltoside at a final concentration of 0.005% (wt/vol). Copper C-flat holey carbon-coated grids (CF-1.2/1.3 300 mesh; EMS) were glow discharged using a PELCO easiGlow before the addition of 3 µl of Fab–trimer complexes. Samples were vitrified in liquid ethane using a Vitrobot Mark IV with a wait time of 30 s and a blot time of 3 s at room temperature with 100% humidity.

### Cryo-EM data collection and processing

Cryo-EM data were collected on an FEI Titan Krios electron microscope operating at 300 kV, equipped with a Gatan K3 direct detector operating in counting mode. Data were acquired using Leginon (Suloway et al., 2005) at 105,000× magnification with a 0.83-Å pixel size and defocus range of 0.8–1.8 µm. A total exposure dose of 58 e^−^/Å^2^ was fractionated over 50 raw frames. All data processing—including motion correction, CTF estimation, particle extraction, 2D classification, ab initio model generation, and 3D refinements—was performed using cryoSPARC (Punjani et al., 2017). All homogenous and nonuniform 3D refinements were performed with C1 symmetry. With the exception of V033-a.01, all other rhesus lineages exhibited 1:1 stoichiometry of trimer–Fab binding. V033-a.01 was observed to bind with 1:1, 1:2, and 1:3 stoichiometries in 2D classes, and we obtained 3D reconstructions ranging from 3.1- to 3.6-Å resolution for each. The varied Fab-binding stoichiometries are likely a result of the limiting 1:3 M ratio of trimer to Fab used to prepare complexes for data collection and not inherent to the biology of V033-a.01.

### Atomic model building

The initial models for all Fab variable regions (Fv) were obtained using the AbodyBuilder2 application of the SAbPred Antibody Prediction Toolbox (Dunbar et al., 2016). The initial models for all Envs were obtained from the Protein Data Bank (PDB) and are specified in Table S6. For each sample, the respective Fv and envelope trimer models were fit into the cryo-EM 3D reconstruction density using UCSF Chimera (Pettersen et al., 2004) or ChimeraX (Meng et al., 2023) to provide an initial model of the Fab–trimer complex. Each structure was solved using iterative rounds of manual model rebuilding in Coot (Emsley and Cowtan, 2004) and automated real-space refinement of the model in Phenix (Liebschner et al., 2019). Overall structure quality was periodically determined using MolProbity (Chen et al., 2010) and EMRinger (Barad et al., 2015) until satisfactory validation of the model was achieved. Fab-interactive surfaces were analyzed using PDBePISA (Krissinel and Henrick, 2007). Summaries of cryo-EM data collection, 3D reconstruction, and model refinement statistics for each structure are provided in Table S6 and Data S1.

### Antibody angles of approach and binding orientation

The latitudinal angles of the antibody approach to the V2 apex of the Env were calculated with UCSF ChimeraX (Meng et al., 2023). The latitudinal access of a V2 apex–targeted antibody is the freedom between the threefold trimer axis and the V2 apex plane, the latter of which we define as the horizontal plane passing through the Cα-atoms of C-strand Lys168 residues on all three protomers. Since the rhesus and human antibodies analyzed in this study all utilize HCDR3-dominated modes of recognition, and these HCDR3s all adopt pronounced conformations extending from the Fab combining surface, we next defined the axis of the antibody as the long axis of the HCDR3. The HCDR3 axis is the vector connecting the centers (centroids) of the Fv itself and the HCDR3, which are defined as the averaged coordinates of Cα-atoms of the two pairs of Fv conserved MolProbity and the averaged coordinates of heavy chain residues 93 and 102 (Kabat numbering), respectively. The angle of intersection between the V2 apex plane and the antibody axis was calculated with the built-in function of ChimeraX. To compare the relative orientations of rhesus and human antibody heavy and light chains, alignments were made with gp120 from each trimer complex (residues 128–192 for the CH03–V1V2 scaffold complex [PDB ID 5ESV]) utilizing the same mode of V2 apex recognition using MatchMaker in ChimeraX.

### Analysis of Indian rhesus macaque peripheral DH3 gene–derived naïve B cells

The B cell repertoires for Indian rhesus macaque DH3 family gene frequency and charge were downloaded from NCBI under accession numbers ERR4250665 to ERR4250672. Germline V, D, and J genes were obtained from the KIMDB 1.1 database (Vázquez Bernat et al., 2021). We processed the BCR transcripts using the SONAR version 2.0 bioinformatics pipeline (Schramm et al., 2016), which includes steps for quality control and annotation. V(D)J gene assignment for each transcript was performed using BLASTn (Ye et al., 2013) (https://www.ncbi.nlm.nih.gov/igblast/index.cgi), with customized parameters against the germline gene database from KIMDB. CDR3 identification was based on BLASTn alignments of the V and J regions, utilizing the conserved second cysteine in the V region and the WGXG (heavy chain) or FGXG (light chain) motifs in the J region, where “X” represents any amino acid. For heavy chain transcripts, isotype was assigned using the constant domain 1 (CH1) sequences through BLASTn against a database of rhesus CH1 genes from IMGT, with a BLAST E-value threshold of 10^(−6) to determine significant isotype assignments. The CH1 allele with the lowest E-value was selected. Non-V(D)J sequences were removed, and transcripts with incomplete or frameshifted V(D)J regions were excluded. Remaining transcripts were aligned to their assigned germline V and D genes using Clustal Omega (Sievers et al., 2011). Frequencies, lengths, and net charges of DH3-derived transcripts were then calculated using custom Python scripts.

### Mass spectrometry to quantify tyrosine sulfation

Each full-length IgG1 antibody was diluted to ∼3 mg/ml using 50 mM ammonium bicarbonate (Thermo Fisher Scientific). For IdeS digestion and deglycosylation, 8 µl of the antibody solution was placed in a microcentrifuge tube. 1 µl of IdeS (40 U/µl, Promega) was added to the antibody solution and incubated at 37°C for 1 h 2 µl of Rapid PNGase F Buffer (NEB) was added and incubated at 80°C for 3 min. The solution was cooled, and 1 µl of Rapid PNGase F was added and incubated at 50°C for 30 min. After deglycosylation, 40 µl of 50 mM ammonium bicarbonate was added prior to LC-MS analysis. For LC-MS analysis, 3–10 µl of the IdeS-digested and deglycosylated sample was injected onto a Waters H-class UPLC, separated with a C4 column (Acquity UPLC Protein BEH C4 column, 300 Å, 17 µm, 2.1 × 100 mm, Waters) set to 80°C and with a flow rate of 0.3 ml/min. Mobile phase A was water with 0.1% formic acid (Thermo Fisher Scientific), and mobile phase B was acetonitrile with 0.1% formic acid (Thermo Fisher Scientific). The gradient was as follows: 0 min, 10%B; 3 min, 10%B; 3.1 min, 25%B; 33 min, 45%B; 33.1 min, 95%B; 36 min, 95%B; 36.1 min, 10%B; and 40 min, 10%B. The column eluate was analyzed by mass spectrometry (Xevo TQ-S, Waters, or Q Exactive HF, Thermo Fisher Scientific). Mass spectra were processed with Mass Lynx (Waters Xevo data) or BioPharma Finder (Thermo Fisher Scientific QE-HF data). Three antibodies (42056-a.01, 44715-a.01, and 6561-a.01) had as many as four sulfation proteoforms detected per digested F(ab’)^2^ molecule, indicating two sites of tyrosine sulfation on each Fab. For 6561-a.01, we modeled both sulfated tyrosines at positions Y100b_HCDR3_ and Y100f_HCDR3_. For 44715-a.01, we modeled just one sulfated tyrosine at position Y100f_HCDR3_ but suspect the second sulfation modification to occur at adjacent residue position Y100g_HCDR3_. For 42056-a.01, we modeled just one sulfated tyrosine at position Y100c_HCDR3_ but suspect the second sulfation modification to occur at adjacent residue position Y100d_HCDR3_. We unexpectedly found many *O*-linked glycoforms for each rhesus antibody irrespective of the presence or absence of tyrosine sulfation, but not for the human antibody controls PGDM1400 and ACS202. There was a trend for tyrosine sulfation to be increased (both the number of sulfation groups and relative intensity of mass peaks) in the presence of *O*-linked glycosylation for the rhesus antibodies.

### Quantification and statistical analysis

The difference in mean values for distributions of frequency, HCDR3 length, and HCDR3 net charge for DH3 family derived naïve rhesus B cells was calculated with Student’s *t* test. The difference in mean values for Fab–V2 apex interactive surface features grouped by species and mode of recognition was calculated using unpaired *t* test. These statistical analyses were performed using GraphPad Prism 10.

### Online supplemental material

Fig. S1 shows the identification of SHIV-infected rhesus macaques with V2 apex–targeted heterologous neutralization breadth and provides examples of flow cytometry gating strategy for antigen-specific memory B cell sorting to isolate mAbs. Fig. S2 provides the analysis of mAb neutralization data to characterize phenotypic properties and determine the extent of recapitulation of polyclonal plasma activity. Fig. S3 shows the human and rhesus macaque immunoglobulin sequence analysis to identify non-templated HCDR3 insertions, determine homologous broadly neutralizing antibody DH genes of interest in each species, and predict the propensity for DH genes to be tyrosine sulfated. Fig. S4 shows the results of mass spectrometry analysis to identify tyrosine sulfation and *O*-linked glycosylation in human and rhesus broadly neutralizing antibodies. Fig. S5 shows the structural and chemical definition of rhesus V2 apex broadly neutralizing antibody classes and identifies one that is multi-donor (RHA1-class). Table S1 provides the neutralization data for plasma and mAbs against small and large panels of heterologous viruses. Table S2 provides the macaque host information, isolation strategy, and immunogenetics for V2 apex lineages. Table S3 provides the results of naïve B cell repertoire sequence analysis for all macaque hosts in this study. Table S4 provides the neutralization data for V2 apex epitope mapping and to demonstrate recapitulation of macaque polyclonal plasma activity by isolated antibodies. Table S5 provides the information for cryo-EM data acquisition, processing, and structure validation statistics. Table S6 provides the structural analysis of human and rhesus V2 apex broadly neutralizing antibodies in complex with HIV Env.

## Supplementary Material

Table S1 provides the neutralization data for plasma and mAbs against small and large panels of heterologous viruses.

Table S2 provides the macaque host information, isolation strategy, and immunogenetics for V2 apex lineages.

Table S3 provides the results of naïve B cell repertoire sequence analysis for all macaque hosts in this study.

Table S4 provides the neutralization data for V2 apex epitope mapping and to demonstrate the recapitulation of macaque polyclonal plasma activity by isolated antibodies.

Table S5 provides the information for cryo-EM data acquisition, processing, and structure validation statistics.

Table S6 provides the structural analysis of human and rhesus V2 apex broadly neutralizing antibodies in complex with HIV Env.

Data S1 provides cryo-EM data processing validation for 3D reconstructions and their corresponding structures.

## Data Availability

Rhesus V, D, and J genes sequenced and identified from naïve B cells in this study were deposited in GenBank and can be accessed using the following GenBank accession numbers: PP648252–PP649987. The variable heavy and light chain gene sequences for recombinant mAbs were deposited in GenBank and can be accessed using the following GenBank accession numbers: PP909817–PP910010. Rhesus B cell Illumina next-generation sequencing datasets are deposited in NCBI Sequence Read Archive under BioProject accession number: PRJNA1121265. The atomic models generated during this study are available at PDB (PDB, https://www.rcsb.org) under the following PDB accession codes: 9BNK, 9BNL, 9BNM, 9BNP, 9BTH, 9BTI, 9BTJ, 9BTL, and 9BTV. The corresponding cryo-EM reconstructions generated during this study are available at the Electron Microscopy Data Bank (EMDB, https://www.ebi.ac.uk/emdb/) under the following EMDB access codes: 44728, 44729, 44730, 44733, 44890, 44891, 44892, 44893, and 44897.
