# Supplementary material for: Structural and genetic basis of HIV-1 envelope V2 apex recognition by rhesus broadly neutralizing antibodies
Source: J Exp Med. 2025 Aug 18;222(10):e20250638. doi: 10.1084/jem.20250638 (PMC12379892; doi:10.1084/jem.20250638)
Supplement: Data S1 — provides cryo-EM data processing validation for 3D reconstructions and their corresponding structures. [file jem_20250638_datas1.pdf]

Data S1-Figure 1

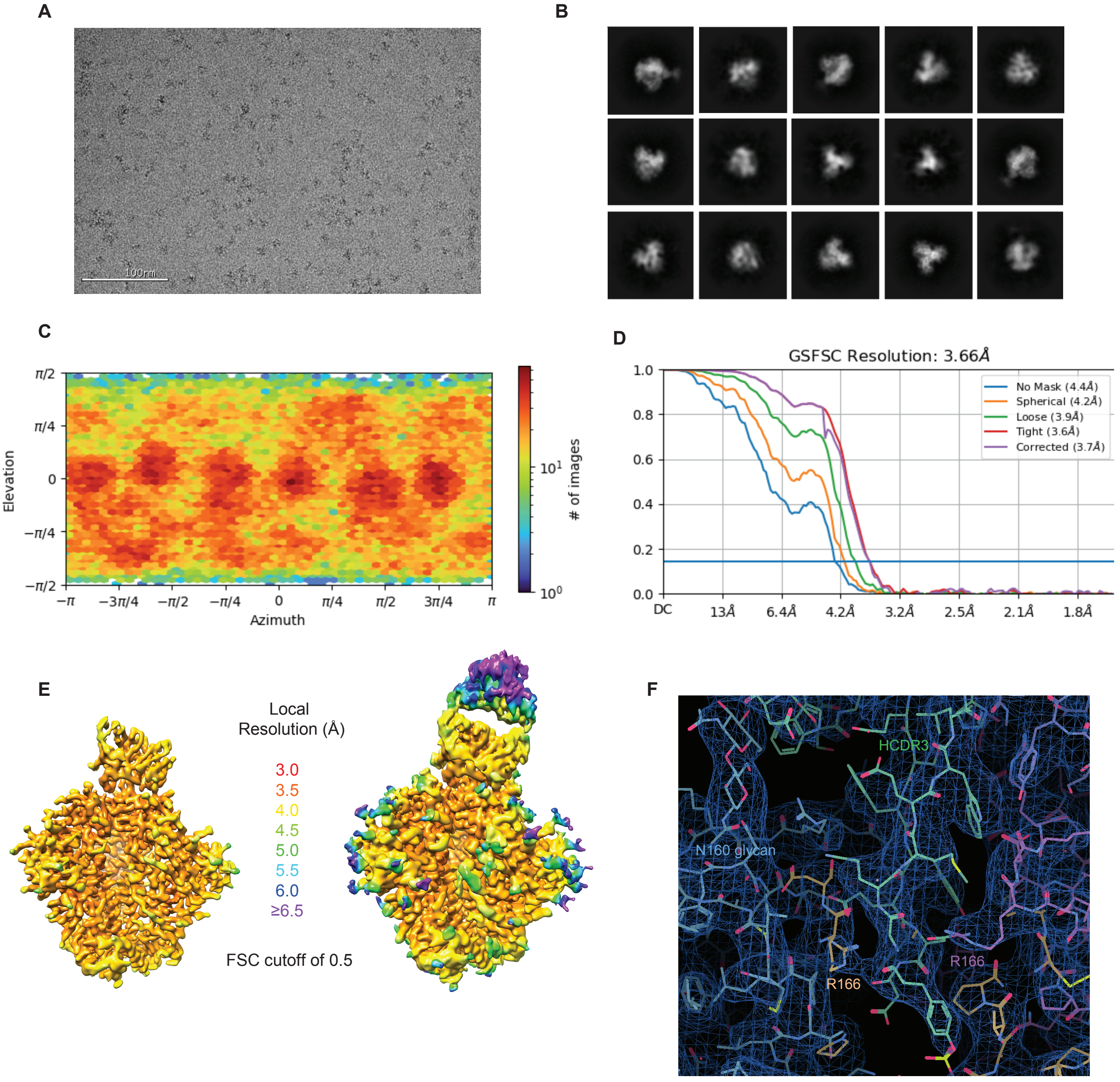

Data S1-Figure 1. **Cryo-EM details of 6070-a.01 in complex with Q23.17 MD39 Env.**
(A) Representative raw micrograph is shown.
(B) Representative 2D class averages of pick particles are shown.
(C) The orientations of all particles used in the final refinement are shown as a heatmap.
(D) The gold-standard fourier shell correlation (FSC) at threshold of 0.143 resulted in a resolution of 3.66 Å using non-uniform refinement with C1 symmetry.
(E) The local resolution of the full map is shown as generated through cryoSPARC using an FSC cut-off of 0.5. Two volume contour levels are shown.
(F) Cryo-EM 3D reconstruction density to highlight the Fab-trimer interactive surface.

Data S1-Figure 2

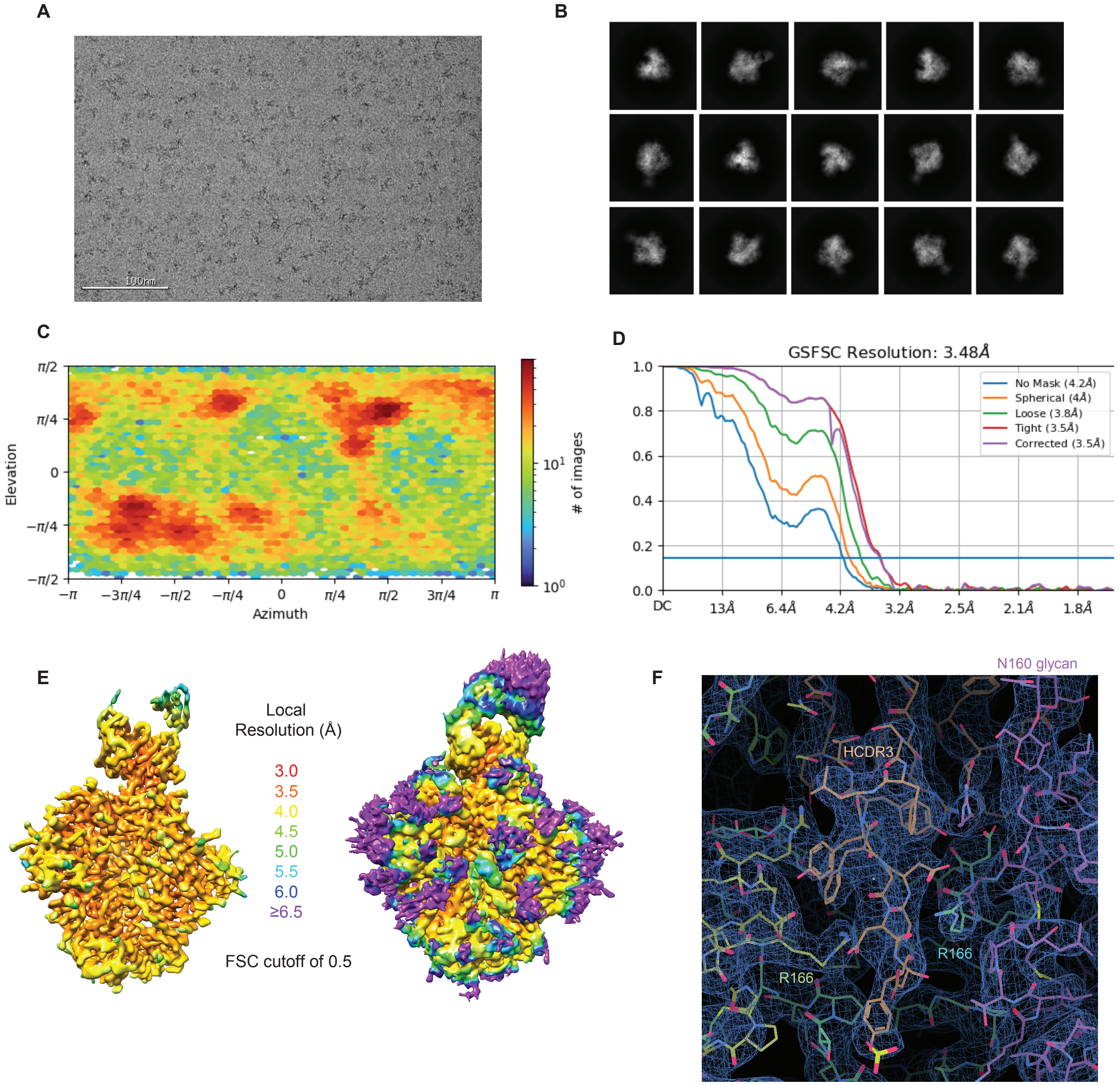

Data S1-Figure 2. **Cryo-EM details of T646-a.01 in complex with Q23.17 MD39 Env.**
(A) Representative raw micrograph is shown.
(B) Representative 2D class averages of pick particles are shown.
(C) The orientations of all particles used in the final refinement are shown as a heatmap.
(D) The gold-standard fourier shell correlation (FSC) at threshold of 0.143 resulted in a resolution of 3.48 Å using non-uniform refinement with C1 symmetry.
(E) The local resolution of the full map is shown generated through cryoSPARC using an FSC cutoff of 0.5. Two volume contour levels are shown.
(F) Cryo-EM 3D reconstruction density to highlight the Fab-trimer interactive surface.

Data S1-Figure 3

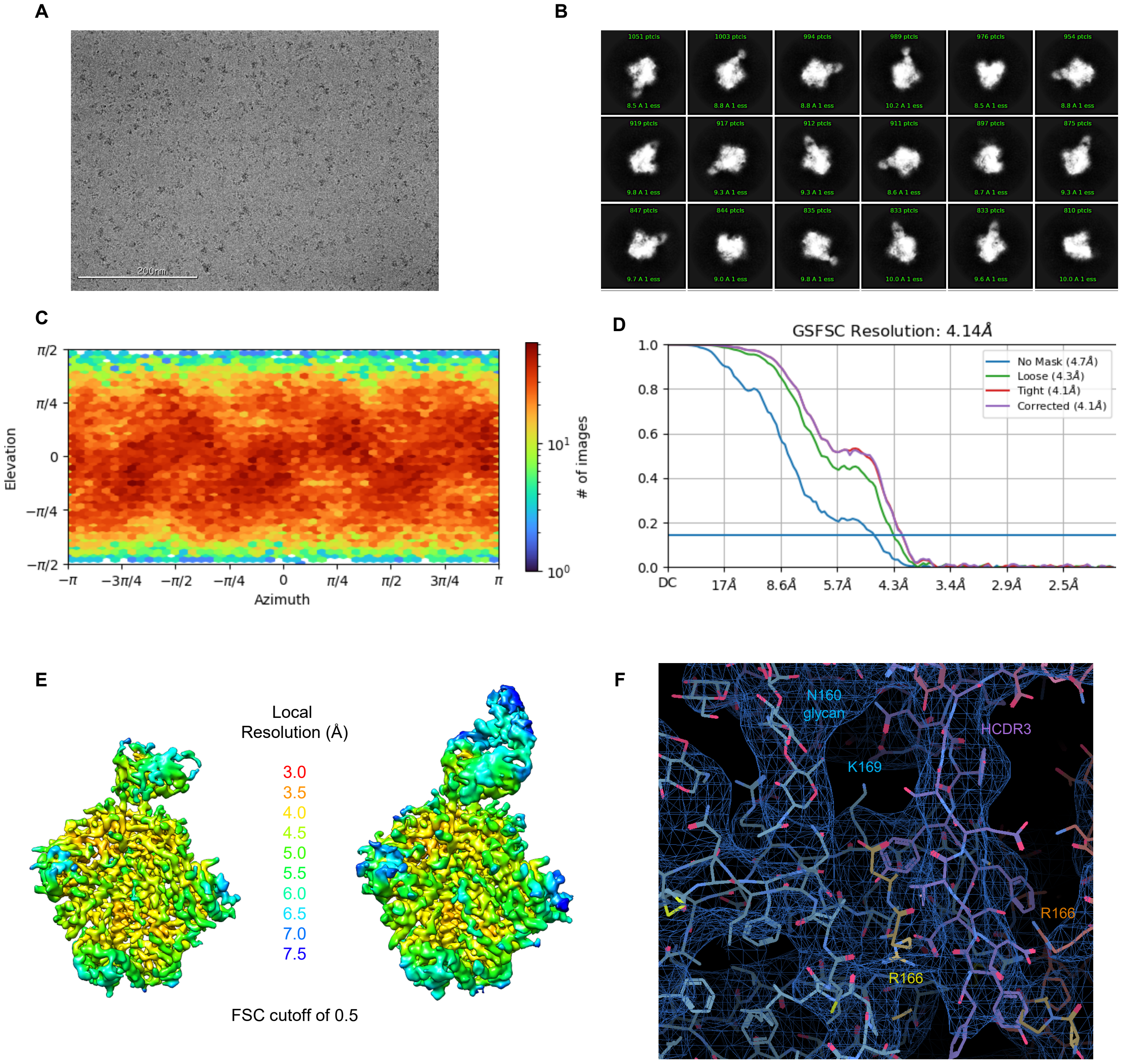

Data S1-Figure 3. **Cryo-EM details of 42056-a.01 in complex with CAP256.wk34.c80 RnS2 SOSIP.**
(A) Representative raw micrograph is shown.
(B) Representative 2D class averages of pick particles are shown.
(C) The orientations of all particles used in the final refinement are shown as a heatmap.
(D) The gold-standard fourier shell correlation (FSC) at threshold of 0.143 resulted in a resolution of 4.20 Å using non-uniform refinement with C1 symmetry; the multimodal behavior of the FSC curve do not appear to be related to masking or local refinement, nor reflected in the quality of the maps.
(E) The local resolution of the full map is shown generated through cryoSPARC using an FSC cutoff of 0.5. Two volume contour levels are shown.
(F) Cryo-EM 3D reconstruction density to highlight the Fab-trimer interactive surface.

Data S1-Figure 4

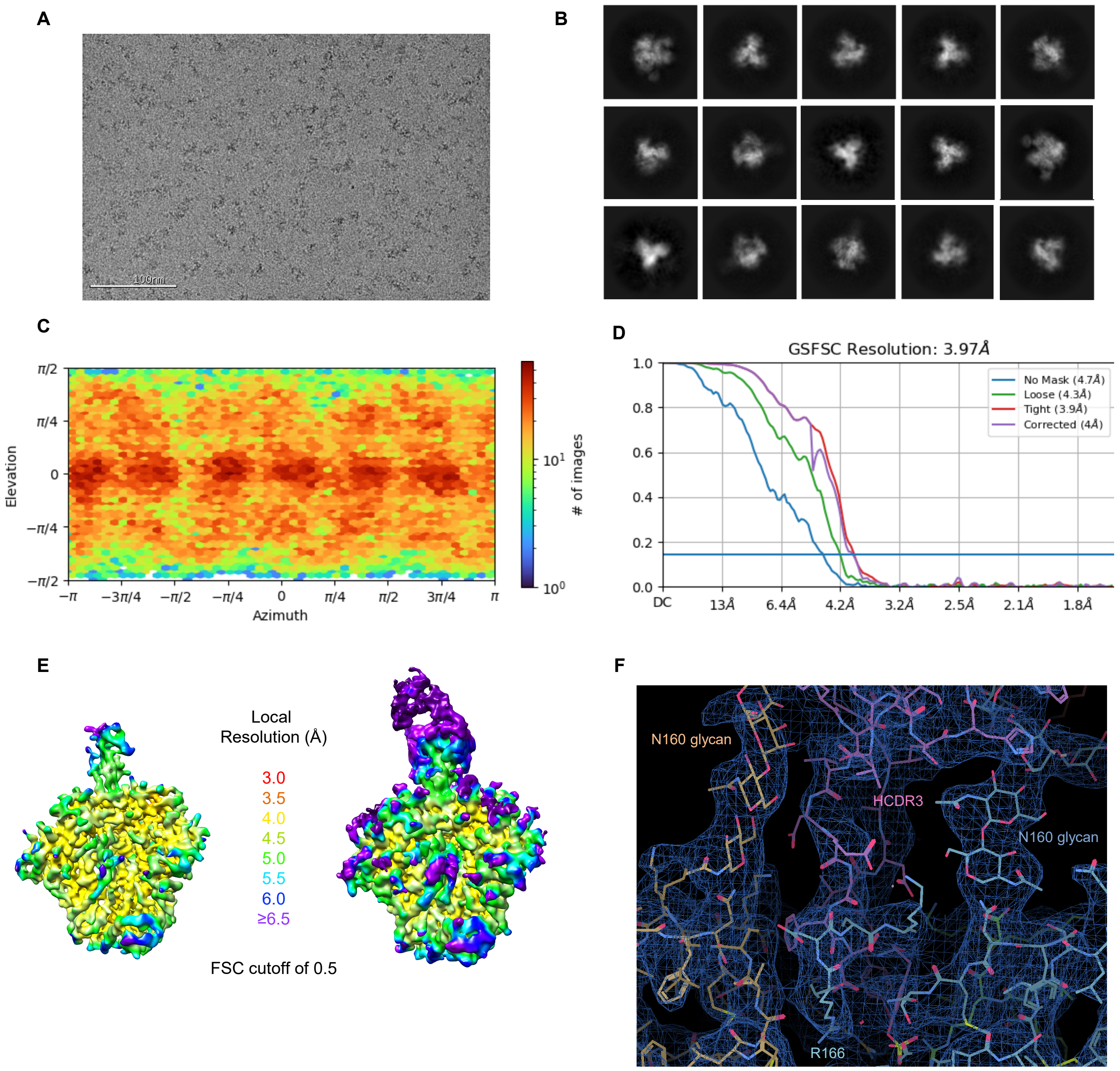

Data S1-Figure 4. **Cryo-EM details of 44715-a.01 in complex with BG505 DS-SOSIP.**
(A) Representative raw micrograph is shown.
(B) Representative 2D class averages of pick particles are shown.
(C) The orientations of all particles used in the final refinement are shown as a heatmap.
(D) The gold-standard fourier shell correlation (FSC) at threshold of 0.143 resulted in a resolution of 3.97 Å using non-uniform refinement with C1 symmetry.
(E) The local resolution of the full map is shown generated through cryoSPARC using an FSC cutoff of 0.5. Two volume contour levels are shown.
(F) Cryo-EM 3D reconstruction density to highlight the Fab-trimer interactive surface.

Data S1-Figure 5

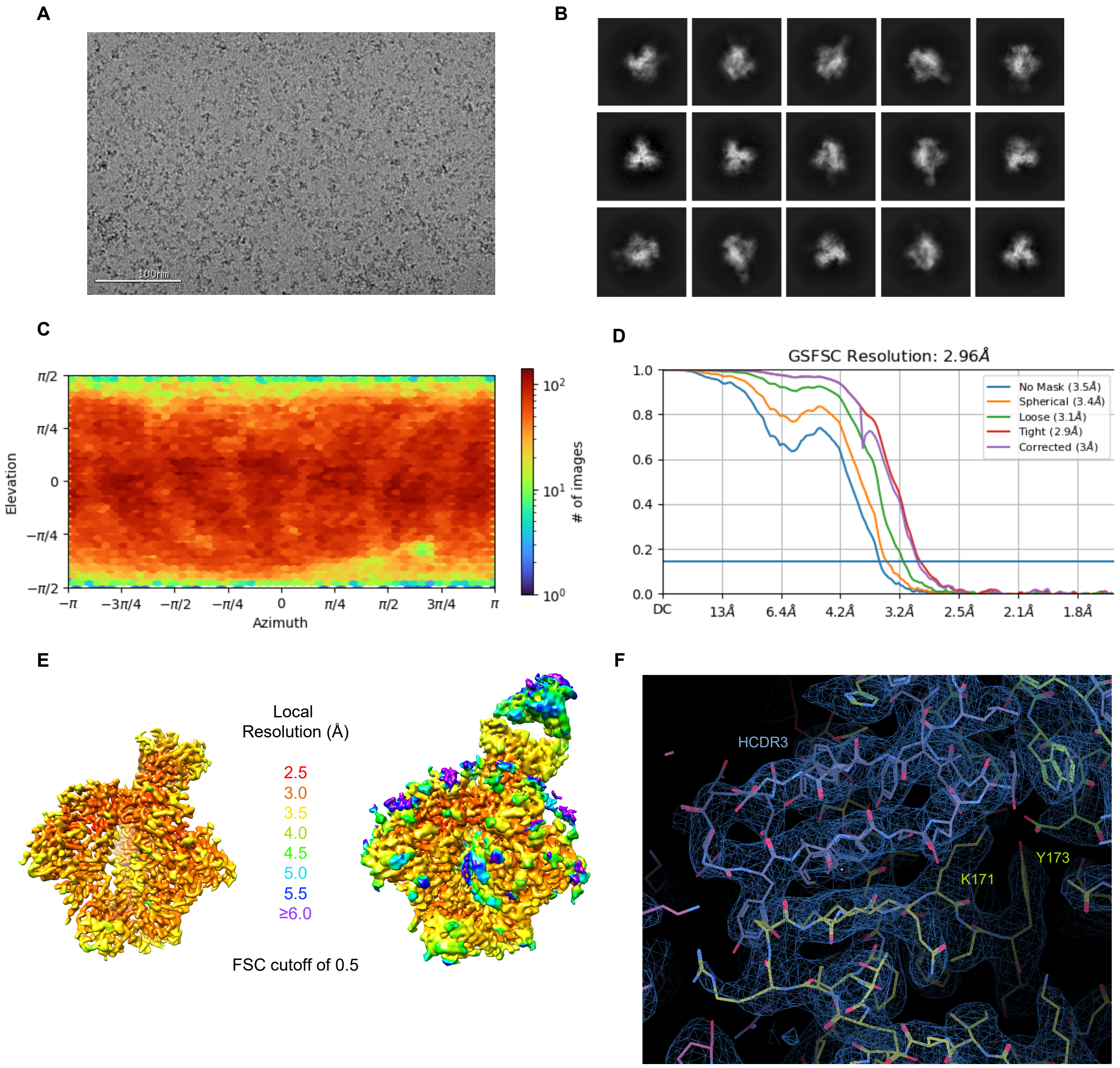

Data S1-Figure 5. **Cryo-EM details of 41328-a.01 in complex with BG505 DS-SOSIP.**
(A) Representative raw micrograph is shown.
(B) Representative 2D class averages of pick particles are shown.
(C) The orientations of all particles used in the final refinement are shown as a heatmap.
(D) The gold-standard fourier shell correlation (FSC) at threshold of 0.143 resulted in a resolution of 2.96 Å using non-uniform refinement with C1 symmetry.
(E) The local resolution of the full map is shown generated through cryoSPARC using an FSC cutoff of 0.5. Two volume contour levels are shown.
(F) Cryo-EM 3D reconstruction density to highlight the Fab-trimer interactive surface.

Data S1-Figure 6

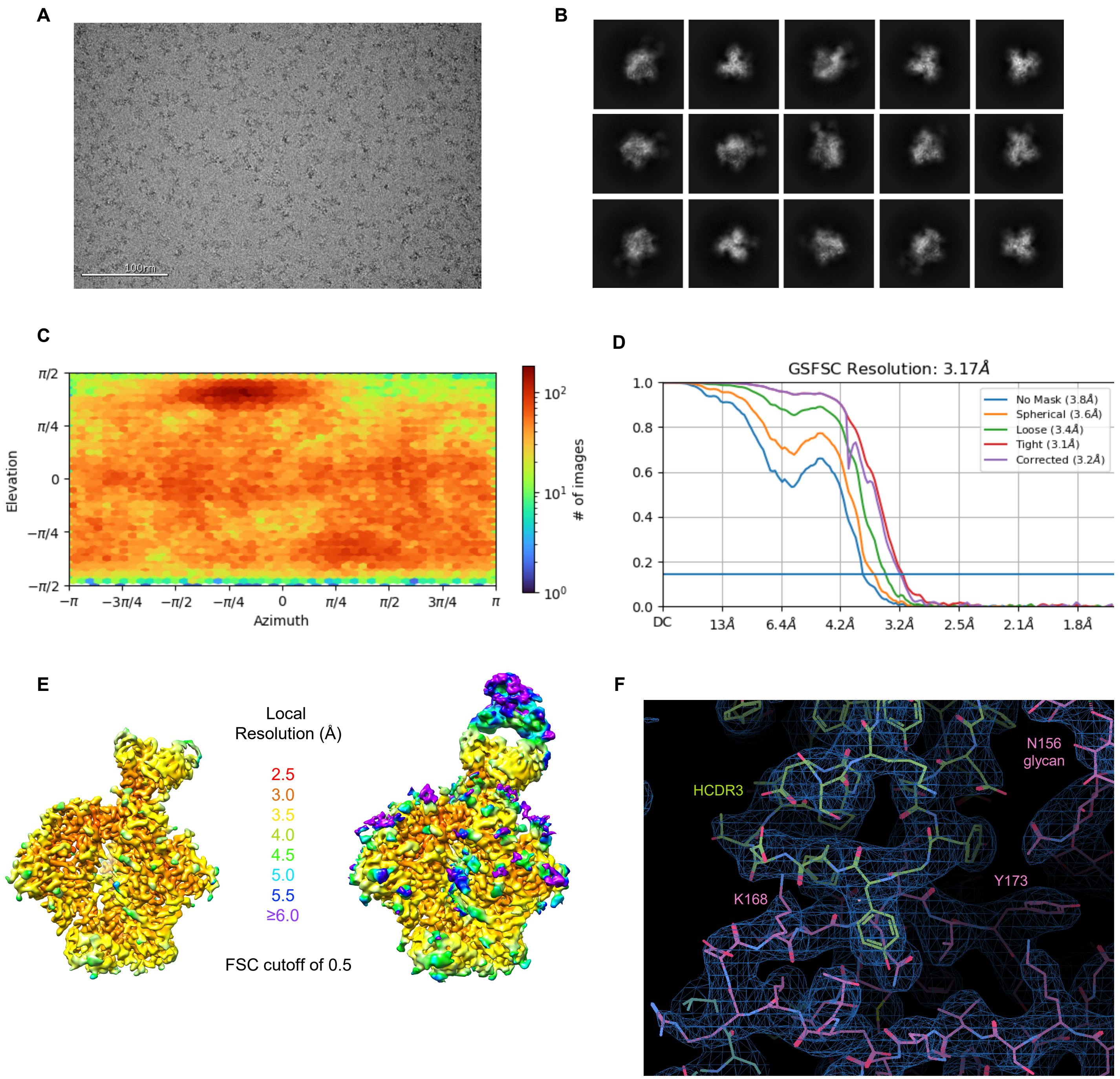

Data S1-Figure 6. **Cryo-EM details of V033-a.01 in complex with BG505 DS-SOSIP.**
(A) Representative raw micrograph is shown.
(B) Representative 2D class averages of pick particles are shown.
(C) The orientations of all particles used in the final refinement are shown as a heatmap.
(D) The gold-standard fourier shell correlation (FSC) at threshold of 0.143 resulted in a resolution of 3.17 Å using non-uniform refinement with C1 symmetry.
(E) The local resolution of the full map is shown generated through cryoSPARC using an FSC cutoff of 0.5. Two volume contour levels are shown.
(F) Cryo-EM 3D reconstruction density to highlight the Fab-trimer interactive surface.

Data S1-Figure 7

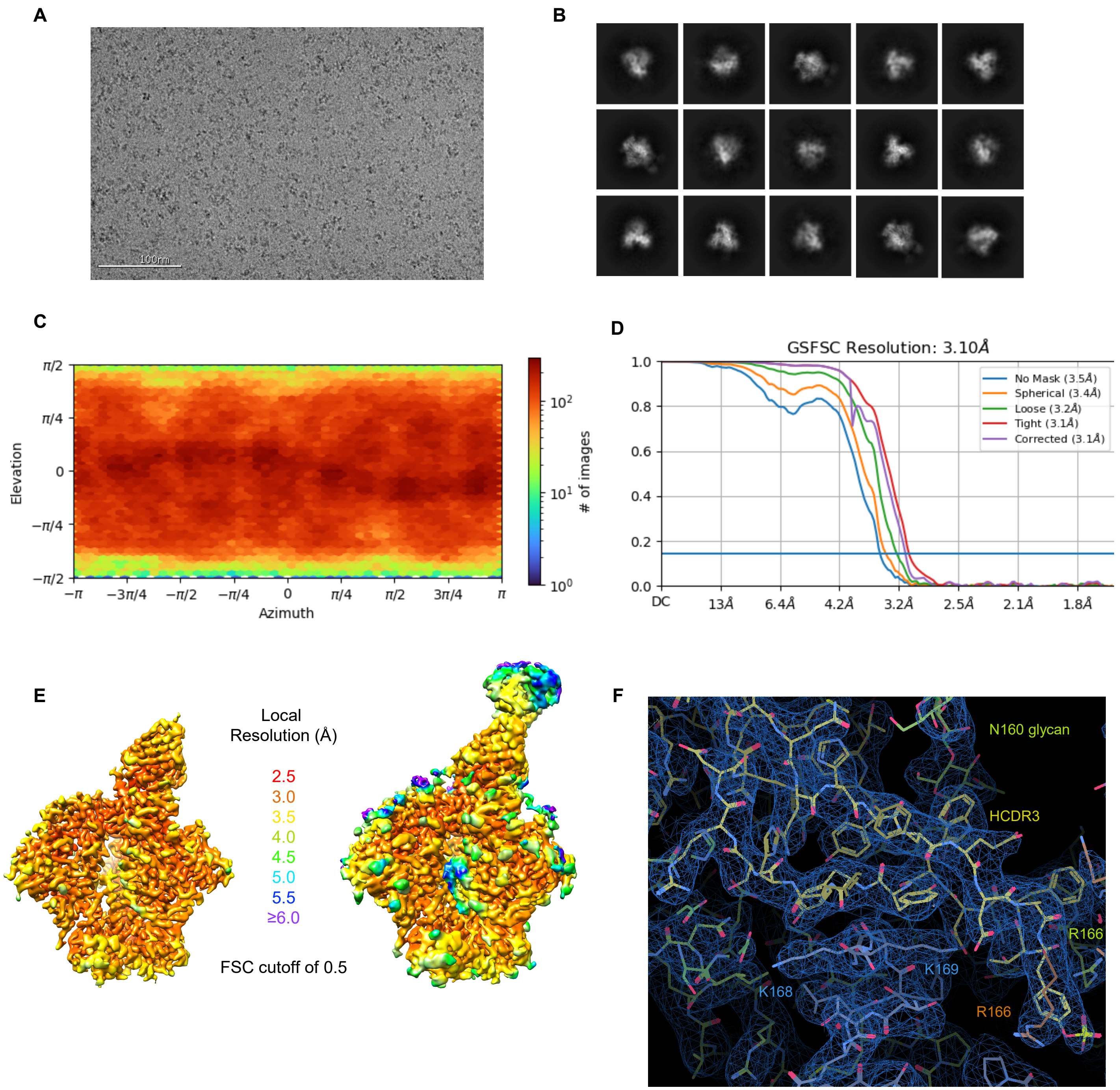

Data S1-Figure 7. **Cryo-EM details of V031-a.01 in complex with BG505 DS-SOSIP.**

(A) Representative raw micrograph is shown.

(B) Representative 2D class averages of pick particles are shown.

(C) The orientations of all particles used in the final refinement are shown as a heatmap.

(D) The gold-standard fourier shell correlation (FSC) at threshold of 0.143 resulted in a resolution of 3.10 Å using non-uniform refinement with C1 symmetry.

(E) The local resolution of the full map is shown generated through cryoSPARC using an FSC cutoff of 0.5. Two volume contour levels are shown.

(F) Cryo-EM 3D reconstruction density to highlight the Fab-trimer interactive surface.

Data S1-Figure 8

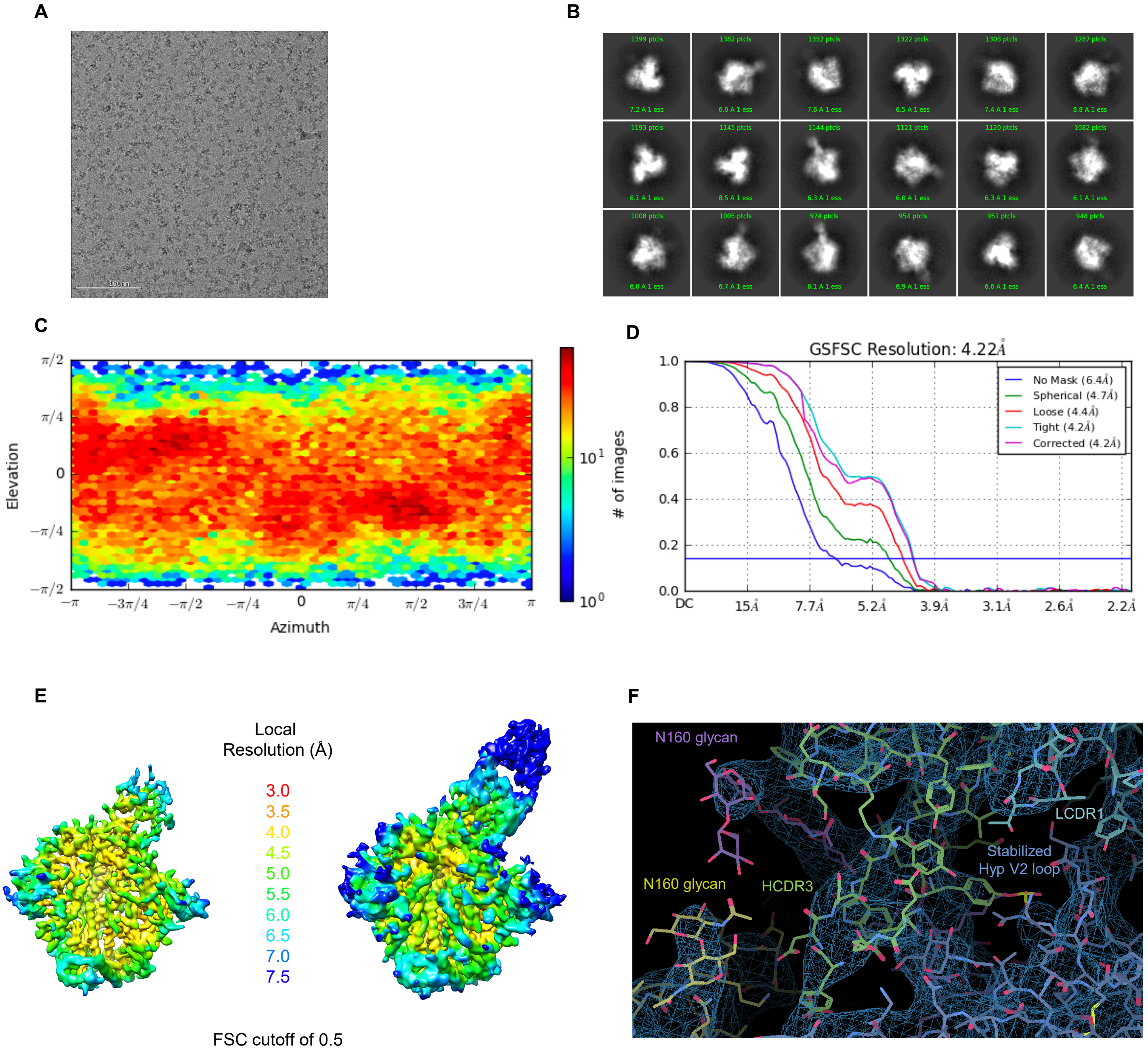

Data S1-Figure 8. **Cryo-EM details of 6561-a.01 in complex with Ce1176 RnS2 SOSIP.**
(A) Representative raw micrograph is shown.
(B) Representative 2D class averages of pick particles are shown.
(C) The orientations of all particles used in the final refinement are shown as a heatmap.
(D) The gold-standard fourier shell correlation (FSC) at threshold of 0.143 resulted in a resolution of 4.22 Å using non-uniform refinement with C1 symmetry; the multimodal behavior of the FSC curve do not appear to be related to masking or local refinement, nor reflected in the quality of the maps.
(E) The local resolution of the full map is shown generated through cryoSPARC using an FSC cutoff of 0.5. Two volume contour levels are shown.
(F) Cryo-EM 3D reconstruction density to highlight the Fab-trimer interactive surface.

Data S1-Figure 9

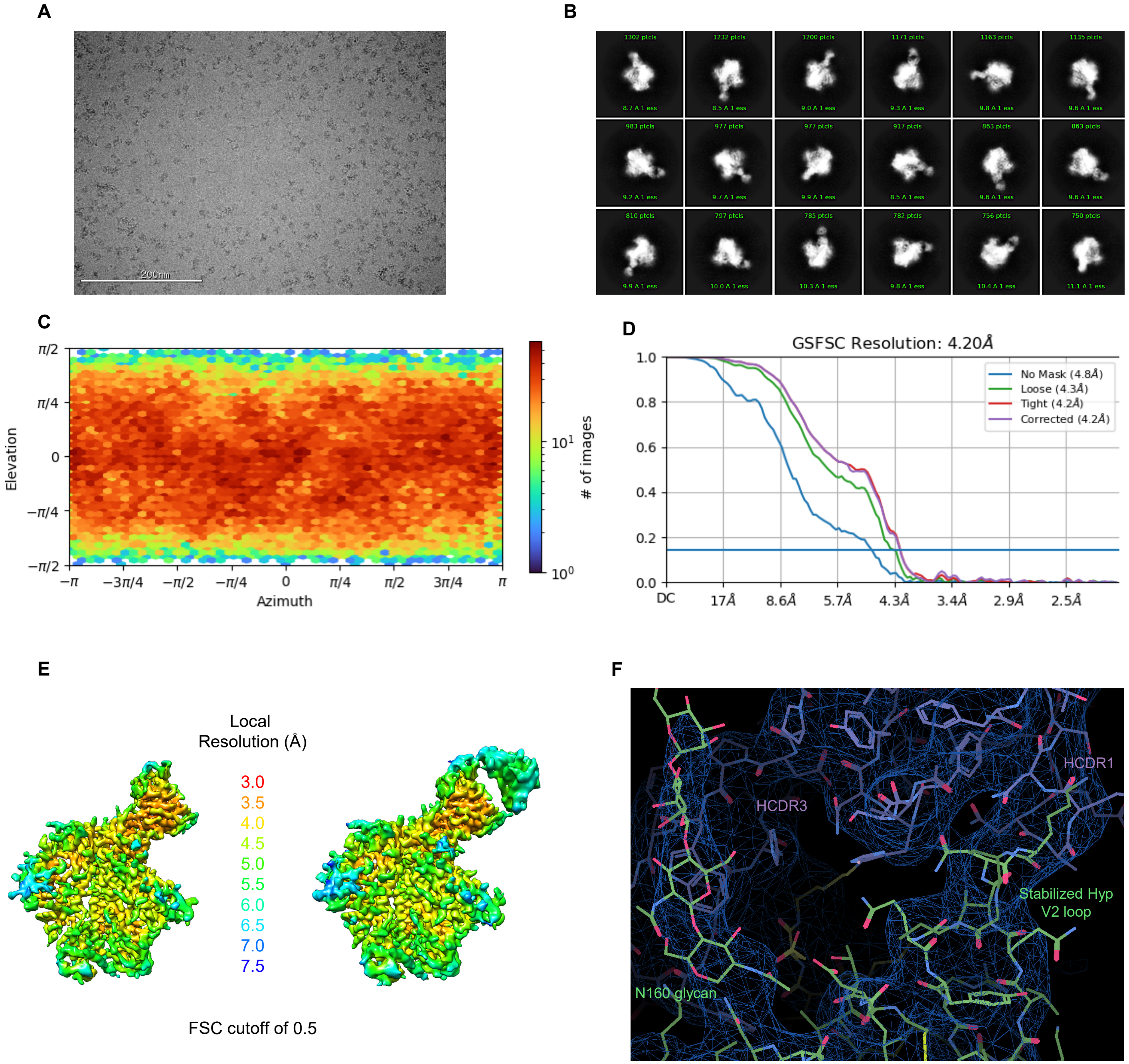

Data S1-Figure 9. **Cryo-EM details of 40591-a.01 in complex with T250.4 RnS2 SOSIP.**
(A) Representative raw micrograph is shown.
(B) Representative 2D class averages of pick particles are shown.
(C) The orientations of all particles used in the final refinement are shown as a heatmap.
(D) The gold-standard fourier shell correlation (FSC) at threshold of 0.143 resulted in a resolution of 4.14 Å using non-uniform refinement with C1 symmetry; the multimodal behavior of the FSC curve do not appear to be related to masking or local refinement, nor reflected in the quality of the maps.
(E) The local resolution of the full map is shown generated through cryoSPARC using an FSC cutoff of 0.5. Two volume contour levels are shown.
(F) Cryo-EM 3D reconstruction density to highlight the Fab-trimer interactive surface.

Data S1-Figure 10

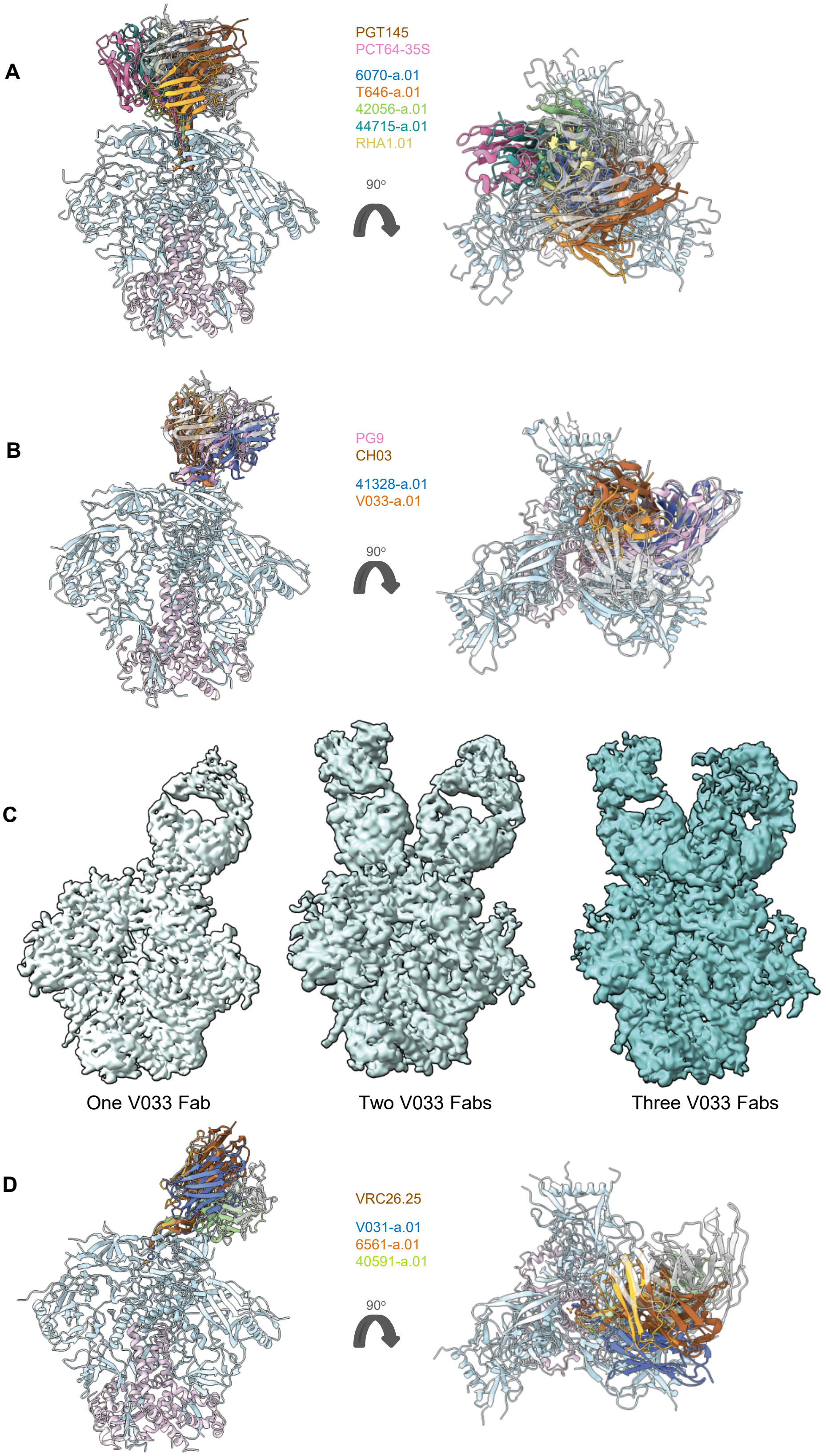

Data S1-Figure 10. **Rhesus and human lineages bind the V2 apex with a constellation of Fab orientations that do not segregate by species.**
(A, B, and D) Structures of Fab complexes with (A) PGT145/needle-like, (B) PG9/axe-like, and (D) VRC26/combined modes of V2 apex recognition are aligned by gp120. Heavy chains are colored according to each respective legend and light chains are shown in transparent light gray.
(C) Cryo-EM 3D reconstruction density with one, two, and three V033-a.01 Fabs bound to BG505 DS-SOSIP Env in the prefusion-closed conformation.
